# Cyclins and CDKs in the regulation of meiosis-specific events

**DOI:** 10.3389/fcell.2022.1069064

**Published:** 2022-11-29

**Authors:** Inés Palacios-Blanco, Cristina Martín-Castellanos

**Affiliations:** Instituto de Biología Funcional y Genómica (IBFG), CSIC-USAL, Salamanca, Spain

**Keywords:** meiosis, prophase, cyclins, CDKs, nuclear architecture, recombination, synapsis, substrates

## Abstract

How eukaryotic cells control their duplication is a fascinating example of how a biological system self-organizes specific activities to temporally order cellular events. During cell cycle progression, the cellular level of CDK (Cyclin-Dependent Kinase) activity temporally orders the different cell cycle phases, ensuring that DNA replication occurs prior to segregation into two daughter cells. CDK activity requires the binding of a regulatory subunit (cyclin) to the core kinase, and both CDKs and cyclins are well conserved throughout evolution from yeast to humans. As key regulators, they coordinate cell cycle progression with metabolism, DNA damage, and cell differentiation. In meiosis, the special cell division that ensures the transmission of genetic information from one generation to the next, cyclins and CDKs have acquired novel functions to coordinate meiosis-specific events such as chromosome architecture, recombination, and synapsis. Interestingly, meiosis-specific cyclins and CDKs are common in evolution, some cyclins seem to have evolved to acquire CDK-independent functions, and even some CDKs associate with a non-cyclin partner. We will review the functions of these key regulators in meiosis where variation has specially flourished.

## Introduction

Meiosis is the cell division program that ensures sexual reproduction by the generation of haploid gametes from diploid cells. Different meiosis-specific hallmarks help to the accurate partitioning and shuffling of the genetic information, which is essential for the viability of gametes, and the efficient transmission and variability of genomes from one generation to the next. These characteristics range from a new nuclear architecture, the formation of a highly organized zipper-like structure between homologous chromosomes (synaptonemal complex, SC) and the promotion of homologous recombination, to the mono-orientation of sister-kinetochores and the sequential degradation of sister-chromatid cohesion (Petronczki et al., 2003; Marston and Amon, 2004; Sakuno and Watanabe, 2009; Keeney et al., 2014; Lam and Keeney, 2014; Zickler and Kleckner, 2015). As a result of these new meiotic features, gametes receive the correct number of chromosomes that after fertilization will ensure the maintenance of species ploidy. Errors during meiosis produce gametes with an abnormal number of chromosomes (aneuploidy), that in some species such as mammals (specially in humans) are very frequent and the leading cause of miscarriages (Hassold et al., 2007; Hunt and Hassold, 2010; Nagaoka et al., 2012). In addition, deregulation of meiotic genes in somatic cells is a common feature of cancer cells, probably contributing to their characteristic genomic instability (Tuna et al., 2009; Folco et al., 2017; Sou et al., 2022).

As in the case of the mitotic cell cycle, meiotic progression is driven by kinase activities provided by cyclin-CDK (Cyclin-Dependent Kinase) complexes, a serine/threonine kinase bound to a regulatory cyclin subunit (Morgan, 1995). In unicellular eukaryotes such as yeasts, a unique CDK binds to different cyclins to temporally order the cell cycle phases, ensuring that DNA replication (S-phase) occurs prior to segregation into two daughter cells (M-phase); and in fission yeast, even a single cyclin-CDK complex is sufficient to drive a “minimal” mitotic and meiotic cycle (Stern and Nurse, 1996; Coudreuse and Nurse, 2010; Uhlmann et al., 2011; Gutierrez-Escribano and Nurse, 2015). Notably, early cell cycle substrates are very efficiently phosphorylated and require lower CDK activity than the late targets, what contributes to the sequential ordering of S and M-phases (Swaffer et al., 2016). From this minimal conception of the cell cycle, cyclins and CDKs have evolved and diversified, especially in organisms with a more complex developmental biology as higher eukaryotes, indicating a corresponding expansion of functions (Harashima et al., 2013; Malumbres, 2014). Functional diversification of CDK activities is clearly observed in meiosis, where CDK complexes already present in vegetative cells have acquired novel meiotic functions, and meiosis-specific variants have also emerged. We will review these aspects of cyclins and CDKs in meiosis, focusing on their meiosis-specific functions. The meiotic-progression properties of CDK complexes have been recently reviewed (Chotiner et al., 2019; Li et al., 2019; Palmer et al., 2019; MacKenzie and Lacefield, 2020). Phosphorylation networks conducted by several kinases during meiotic prophase are also reviewed in (Kar and Hochwagen, 2021).

## Meiosis-specific events regulated by cyclins and CDKs

**FIGURE 1:**
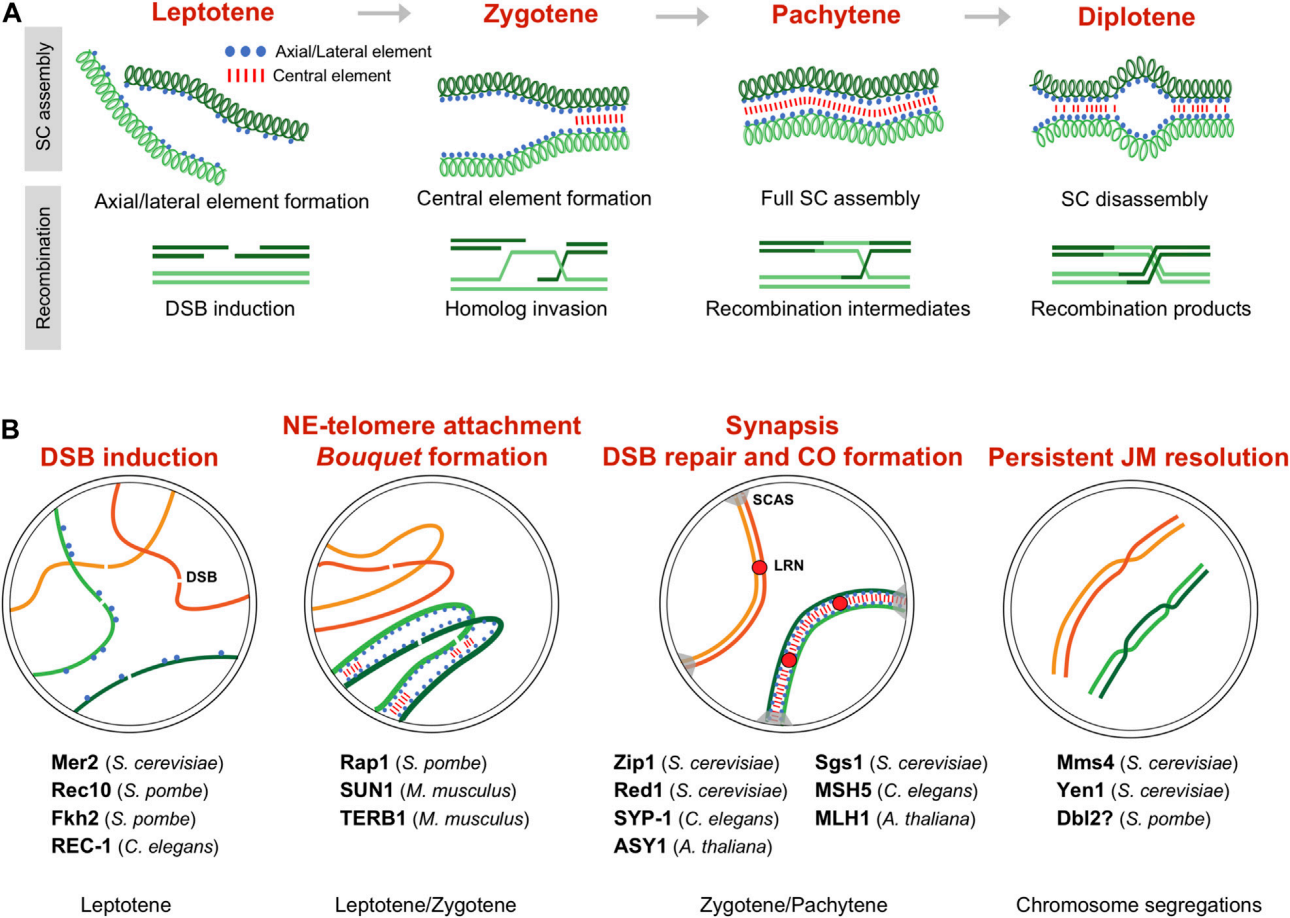
Meiotic prophase and CDK activity. **(A)** Synaptonemal complex (SC) formation and recombination during meiotic prophase between a pair of homologous chromosomes. Prophase is divided in different stages depending on the SC assembly (upper scheme). Recombination occurs in the context of the SC (lower scheme), and, in some organisms, it is linked to SC formation; for simplicity only the chromatid pair involved in recombination is depicted. **(B)** Meiotic events regulated by CDK activity and substrates. The regulated processes during the different prophase stages are highlighted. Identified substrates indicating the species are listed. SC assembly is shown only in one pair of homologs (green colored). DSB double-strand break, SCAS synaptonemal complex attachment sites, LRN late recombination nodule.

A key difference from mitosis is the establishment in meiosis of an extended gap phase (G2), known as meiotic prophase, prior to chromosome segregation. Prophase is cytologically visualized by the remodeling of the nuclear architecture and the formation of the SC or related structures. During prophase, homologous chromosomes align, tightly pair, and recombine. In organisms with a canonical SC, meiotic prophase is divided in different stages depending on the SC assembly; and in budding yeast, mouse, and plants, SC formation and recombination are functionally linked (Padmore et al., 1991; Cohen and Pollard, 2001; Hunter and Kleckner, 2001; Zickler and Kleckner, 2015). Briefly, recombination is initiated by programmed Double-Strand Breaks (DSBs) in the DNA during leptotene, when axial elements of the SC are forming. Processing of these DSBs generates single-stranded DNA nucleofilaments that invade the homologous chromosome searching for homology, which brings homologs closer and promotes local nucleation of the central element of the SC (zygotene). Full SC assembly along the entire chromosome length is the hallmark of pachytene, which correlates with the production of DNA joint molecules between homologs. Finally, at diplotene SC disassembles and resolution of the joint molecules generates final recombination products (Figure 1A). As discuss below, CDKs, cyclins, and non-cyclin CDK activators play an important role in these aspects of meiotic prophase (Figure 1B and Table 1).

**TABLE 1:** CDK substrates in meiotic prophase.

Substrate	Organism	Residues	Evidences	Function	References
Mer2	*S. cerevisiae*	S30	*In vitro* kinase assay, SDS-PAGE mobility, CDK inhibition (*cdc28-as1*), *mer2* ^*S30A*^ and *mer2* ^*S30D*^ phosphomutants	DSB formation	Henderson et al. (2006); Wan et al*.* (2008)
Rec10	*S. pombe*	S347, T482, and S529	Mass spectrometry	DSB formation (*rec10* ^*8A*^ no recombination phenotype)	Spirek et al. (2010); Bustamante-Jaramillo et al. (2019)
Fkh2	*S. pombe*	S481	*In vitro* kinase assay, SDS-PAGE mobility, EMSA assays, *fkh2* ^*S481A*^ and *fkh2* ^*S481D*^ phosphomutants	Transcriptional activation of mid-meiotic genes	Alves-Rodrigues et al. (2016)
REC-1	*C. elegans*	T39, T96, T160, **S146**, S205, S218, S281, T305	*In vitro* peptide array, *in vitro* kinase assay, CDK inhibition S146 (Roscovitine)	DSB formation (*rec-1* ^*8S/TA*^ and *rec-1* ^*8S/TD*^ loss-of- function phenotype)	Chung et al*.* (2015)
Rap1	*S. pombe*	S213, T378, S422, S513, S549	Mass spectrometry, phosphoaffinity SDS-PAGE mobility	*Bouquet* formation (*rap1* ^*32A*^ and *rap1* ^*32E*^ no phenotype)	Amelina et al. (2015)
SUN1	*M. musculus*	S48	*In vitro* kinase assay	NE-telomere attachment/*Bouquet* formation	Viera et al. (2015); Mikolcevic et al. (2016)
TERB1	*M. musculus*	T647	Mass spectrometry, phospho-specific antibody, CDK inhibition (Roscovitine), *terb1* ^*T647A*^ and *terb1* ^*T647D*^ phosphomutants	NE-telomere attachment/*Bouquet* formation	Huttlin et al. (2010); Shibuya et al. (2014) and Shibuya et al. (2015)
Zip1	*S. cerevisiae*	-	*In vitro* kinase assay, SDS-PAGE mobility, CDK inhibition (*cdc28-as1*), Clb5 and Clb6 dependency	Synapsis (*zip1* ^*4SA*^ no phenotype)	Ubersax et al. (2003); Zhu et al. (2010)
Red1	*S. cerevisiae*	-	Phosphoaffinity SDS-PAGE mobility, CDK inhibition (*cdc28-as1*), Clb5 and Clb6 dependency	Synapsis (*red1* ^*7A*^ no phenotype)	Zhu et al. (2010); Lai et al. (2011)
SYP-1	*C. elegans*	T452	*In vitro* kinase assay, phospho-specific antibody, *syp1* ^*T452A*^ phosphomutant	Synapsis	Brandt et al. (2020)
ASY1	*A. thaliana*	**T142,** T184	Mass spectrometry, *in vitro* kinase assay, *asy1* ^*T142V*^, *asy1* ^*T142D*^ and *asy1* ^*T142V;T184V*^ phosphomutants	Synapsis	Yang et al. (2020)
Sgs1	*S. cerevisiae*	S46, T50, T122, S272, S348, S493, S617	Mass spectrometry (mitosis), *in vitro* kinase assay, SDS-PAGE mobility, CDK inhibition (*cdc28-as1*), *sgs1* ^*9A*^ phosphomutant	DSB Repair	Grigaitis et al. (2020)
MSH-5	*C. elegans*	T1009, T1109, S1278	Mass spectrometry (*in vitro* phosphorylated protein), *in vitro* kinase assay, phospho-specific T1009 antibody, *msh5* ^*13A*^ phosphomutant	CO formation	Haversat et al. (2022)
MLH1	*A. thaliana*	-	*In vitro* kinase assay	CO formation	Wijnker et al. (2019)
Mms4	*S. cerevisiae*	S56	Mass spectrometry (mitosis), phosphoaffinity SDS-PAGE mobility, *mms4* ^*14A*^ phosphomutant	DSB Repair (persistent JM resolution)	Matos et al. (2011)
Yen1	*S. cerevisiae*	S71, S245, S500, S583, S655, S679	Mass spectrometry (mitosis), phosphoaffinity SDS-PAGE mobility, Yen^ON(9A)^ phosphomutant	DSB Repair (persistent JM resolution)	Matos et al. (2011); Blanco et al. (2014); Eissler et al. (2014); Alonso-Ramos et al. (2021)

### Nuclear architecture

A conserved feature of meiosis is the remodeling of the nuclear architecture to acquire the so-called *bouquet* configuration (Bass, 2003; Harper et al., 2004; Tomita and Cooper, 2006; Scherthan, 2007; Klutstein and Cooper, 2014). Telomeres, that during vegetative cycle are scattered around the nuclear periphery, polarize in tight proximity to the nuclear envelope (NE), in some organisms close by or even bound to the centrosome. This reorganization requires telomere anchoring to the Linker Nucleoskeleton and Cytoskeleton (LINC)-complex, which is composed of SUN (Sad1, UNC-84) and KASH (Klarsicht, ANC-1, Syne homology) family proteins. SUN-domain proteins are inserted in the inner nuclear membrane and KASH-domain proteins are inserted in the outer nuclear membrane, interacting in the transluminal space. The binding of telomeres to this complex is mediated by meiosis-specific proteins that provide the connection of chromosomes with the cytoskeleton, and, in doing so, promote chromosomal movements that facilitate proper chromosome alignment and recombination (Hiraoka and Dernburg, 2009; Koszul and Kleckner, 2009; Shibuya and Watanabe, 2014; Link et al., 2015; Shibuya et al., 2015; Fan et al., 2022). Depending on the organisms, the *bouquet* configuration is a transient feature or it can be maintained during the entire meiotic prophase. This is the case of the fission yeast *Schizosaccharomyces pombe* where, in the absence of a canonical SC, *bouquet*-led chromosome movements have acquired a prominent role in chromosome pairing (Ding et al., 2004). In addition, this nuclear configuration seems to play unanticipated functions in centromere maturation and spindle formation (Tomita and Cooper, 2007; Klutstein et al., 2015).

**FIGURE 2:**
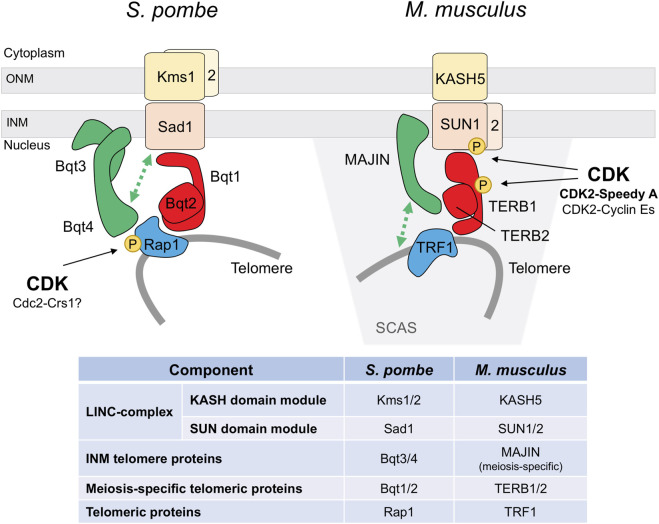
CDK regulation of the meiotic telomere attachment to the nuclear envelope. *S. pombe* and *M. musculus* models. Functional conservation of the different elements is highlighted by the same color. INM inner nuclear membrane, ONM outer nuclear membrane, SCAS synaptonemal complex attachment sites. Protein interactions are based on Chikashige et al., 2006, Chikashige et al., 2009, and Hu et al., 2019 for fission yeast, and Shibuya et al., 2014, Shibuya et al., 2015, and Wang et al., 2020 for mouse.

The striking stable *bouquet* structure and its extremely vigorous motion has made fission yeast an extensively used model to study *bouquet* formation and chromosome movements (Yamamoto and Hiraoka, 2001). In this organism, telomere binding to the Spindle Pole Body (centrosome equivalent) is mediated by pheromone-induced Bqt1 and Bqt2. These proteins connect the conserved telomeric protein Rap1 and the SPB-component Sad1 (SUN-domain protein). This interaction initially delocalizes Sad1 to the scattered telomeres at the NE, and the subsequent travelling of Sad1 back to the SPB promotes the *bouquet* formation (Chikashige et al., 2006; Tomita and Cooper, 2006). Since Sad1 is a NE protein, Bqt1-Bqt2 binding requires previous telomere-NE association, which is mediated by the binding of Rap1 to the NE-anchoring complex Bqt3-Bqt4 during vegetative cell cycle (Chikashige et al., 2009) (Figure 2). Notably, Cdc2 (CDK) and the meiosis-specific Crs1 cyclin localize to the SPB during *bouquet* (Moiseeva et al., 2017; Bustamante-Jaramillo et al., 2021). Crs1 localization is independent of *bouquet* formation, and in the absence of Crs1 the clustering of telomeres is unstable though the integrity of the SPB is preserved (Bustamante-Jaramillo et al., 2021). Although less severe, this phenotype is reminiscent of the defects observed in *bqt1* and *bqt2* mutants. As for *bqt1* and *bqt2*, *crs1* gene expression is induced by pheromone signaling, the protein localizes at the SPB in early prophase, and it remains at SPBs in meiosis I (Chikashige et al., 2006; Tang et al., 2006; Bustamante-Jaramillo et al., 2021). At present, it is not known how Crs1 influences telomere positioning during meiosis and whether it is required for the localization of Bqt1-Bqt2 proteins at the SPB.

Interestingly, Rap1 is highly phosphorylated during meiotic prophase, including at five Cdc2 sites, raising the possibility that CDK phosphorylation could modulate Rap1 interaction with Bqt1-Bqt2 proteins (Amelina et al., 2015) (Figure 2). Three of these sites (Thr378, Ser422, and Ser513) are also specifically phosphorylated at early M-phase in vegetative cell cycle, and Ser513 is critical for releasing telomeres from the NE to facilitate chromosome segregation (Fujita et al., 2012). Cdc2-dependent phosphorylation at these residues weakens the interaction with the NE-anchoring complex Bqt3-Bqt4. Indeed, structural studies support that Ser513 phosphorylation could impair the binding to Bqt4, and the Rap1-S513E mutant protein, mimicking this serine phosphorylation, shows a reduced binding affinity (Hu et al., 2019). Similarly, once the scattered telomeres have bound to Bqt1-Bqt2 and Sad1 in meiosis, Cdc2-dependent Rap1 phosphorylation could diminish the interaction with the Bqt3-Bqt4 complex, facilitating the natural travelling of the telomeres along the NE to gather at the SPB. Otherwise, once the telomeres reach the SPB, local Cdc2-dependent inhibition of the interaction with the Bqt3-Bqt4 complex could enhance Bqt1-Bqt2 binding and reinforce telomere attachment to the SPB. However, *rap1-32A* and *rap1-32E* mutants, in which all the meiosis-identified phosphorylated sites were substituted with non-phosphorylatable residues (alanine) or phosphomimetic residues (glutamic acid), show normal *bouquet* dynamic, and telomeres timely cluster and dissociate from the SPB (Amelina et al., 2015). It is worth noting that these mutants harbor additional mutations apart from the Cdc2-phosphomutant sites, which can obscure the contribution of CDK. Interestingly, by yeast two-hybrid (Y2H) assays the Rap1-32E proteins seems to interact more efficiently with Bqt1-Bqt2 than the wild type protein. In addition, structural studies of a minimal Rap1-Bqt4 complex have identified a Bqt4-binding motif in Rap1 which is also present in other known and newly identified Bqt4-interacting proteins, including Sad1, and these proteins bind to Bqt4 in a competitive manner (Hu et al., 2019). It will be worthy to explore the contribution of Thr378, Ser422, and particularly, Ser513 phosphorylation sites to *bouquet* formation, and their possible regulation by the meiosis-specific Crs1 cyclin.

More recently, telomere anchorage to the NE during meiosis has been also studied in mice, where different components of the LINC-complex and the meiosis-specific linker to telomeres have been identified (Shibuya and Watanabe, 2014; Shibuya et al., 2015) (Figure 2). Interestingly, meanwhile CDK2 is not essential for vegetative growth, it plays a crucial role in meiosis, and knock out (KO) mice are sterile due to spermatocyte and oocyte arrest in prophase and death by apoptosis (Berthet et al., 2003; Ortega et al., 2003). CDK2 localizes to telomeres from leptotene to pachytene (Ashley et al., 2001), and in spermatocytes it is observed in the NE-attachment plates by electron microscopy (Viera et al., 2015). In the absence of CDK2 50% of the telomeres are not properly associated to the NE and the *bouquet* conformation is not observed. *Cdk2* KO spermatocytes present aberrant dynamics in chromosome pairing, with frequent unsynapsed regions, non-homologous associations, and formation of chromosome rings (Viera et al., 2009; Viera et al., 2015). These phenotypes are also observed in *Speedy A* and *cyclin E* mutants.

Speedy A, also known as Ringo A, is a non-cyclin CDK interactor that seems to act as the main activator for CDK2 in telomere dynamics*.* The protein is specifically expressed in the adult testis, being present in spermatocytes from preleptotene to pachytene, and in embryonic ovary when meiotic prophase I occurs (Tu et al., 2017). Speedy A colocalizes with CDK2 in NE-associated telomeres and is essential for the telomeric recruitment of CDK2; indeed, a CDK2 protein carrying mutations on the key residues for Speedy A binding does not localize to the telomeres (Mikolcevic et al., 2016; Tu et al., 2017). The interaction between these proteins is also supported by co-immunoprecipitation and, moreover, by the fact that CDK2 kinase activity is approx. 70% decreased in *Speedy A* KO testis (Mikolcevic et al., 2016; Tu et al., 2017). Similarly to the *Cdk2* KO, lack of Speedy A also impairs homologous pairing and telomere dynamics, showing NE-unattached telomeres inside the nucleus and telomere fusions (Mikolcevic et al., 2016; Tu et al., 2017). Specifically, the telomeric cap-remodeling observed at late prophase is defective in the mutant, and telomeres do not show the characteristic TRF2 (shelterin core component) redistribution into a ring structure (Shibuya et al., 2015; Tu et al., 2017; Chen et al., 2021). Interestingly, Speedy A reaches the telomeres prior to CDK2 and harbors a telomere localization domain that is sufficient to restore the telomere-NE interactions in *Speedy A* KO spermatocytes, indicating a docking function beyond its known CDK2-activation function (Tu et al., 2017).

The mechanism underlying the CDK2-Speedy A essential role in telomere-NE attachment and *bouquet* formation is not completely elucidated. SUN1 is a component of the LINC complex located at the telomere attachment plates associated with the inner nuclear membrane (Ding et al., 2007; Link et al., 2014). In *Cdk2* and *Speedy A* KO mutants, SUN1 protein is delocalized from these spots and observed as a polarized cap-shaped signal at the NE. Lack of SUN1 interactions at the NE could explain the telomere dynamics defects observed in these mutants (Viera et al., 2015; Mikolcevic et al., 2016; Tu et al., 2017). Indeed, SUN1 and CDK2-Speedy A directly interact, and a SUN1 mutant protein in the Speedy A-binding domain exhibits similar phenotypes to those of *Speedy A* and *SUN1* mutants with approx. 50% of the telomeres unattached to the NE (Wang et al., 2020; Chen et al., 2021). Moreover, *in vitro* experiments showed that SUN1 is phosphorylated by CDK2-Speedy A, at least on Ser48 (Viera et al., 2015; Mikolcevic et al., 2016). SUN1 phosphorylation by CDK2-Speedy A could be an important regulatory step to control telomere-NE interactions, and their proper diffusion along the NE necessary for telomere dynamics and *bouquet* formation (Figure 2). Recent studies support this notion (see below).

Cyclin E1 and cyclin E2 also contribute to maintain the integrity of the telomeres and ensure their attachment to the NE in meiosis (Martinerie et al., 2014; Manterola et al., 2016). Although they are not essential for vegetative growth and both KO mice are viable, *E2* KO males are subfertile. In addition, the meiotic phenotypes of *E2* KO mice are enhanced when cyclin E1 levels are disminished, and *E1* ^*+/-*^ *E2* ^*−/−*^ male mice, which reduce cyclin E in *E2 KO* background, are infertile (Martinerie et al., 2014). Deficiency of *E1* and *E2* cyclins in spermatocytes reduces localization of components of the shelterin complex to the chromosome ends. This defect correlates with an increased amount of the DNA-damage γH2AX marker at telomeres, indicating a loss of telomere protection (Manterola et al., 2016). Cyclin E1 and E2 are important for the formation of the synaptonemal complex attachment sites (SCAS, expansions of chromosome ends reflecting the formation of the attachment plates), which are crucial for the stable association of telomeres to NE. Spermatocytes depleted in E1 and E2 cyclins develop narrower SCAS and, in fact, telomere attachment to the NE is compromised since the TRF1 telomere marker (shelterin core component) is detected inside the nuclear space in 47% of the spermatocytes (Martinerie et al., 2014; Manterola et al., 2016). In correlation with these alterations, lack of E2 cyclin causes telomeric and synaptic abnormalities that are enhanced in *E1* ^*+/−*^ *E2* ^*−/−*^ spermatocytes. Although E-type cyclins do not show specific telomeric enrichment, they are likely to act in combination with CDK2 given the similar defects in telomere-NE attachment and synapsis observed in the mutants; particularly the presence of residual membranes in the detached telomeres is observed in both mutants (Viera et al., 2015; Manterola et al., 2016). Indeed, E-type cyclins are necessary for the telomeric localization of CDK2. In the absence of E2 approx. 40% of telomeres exhibit a reduced CDK2 loading, raising up to 93% in *E1* ^*+/−*^ *E2* ^*−/−*^ mice. Moreover, co-immunoprecipitacion analysis confirm that both cyclins interact with CDK2 in spermatocytes *in vivo* (Martinerie et al., 2014).

Defects in shelterin complex integrity at the telomeres in E-cyclin deficient mutants might affect cap-remodeling. In fact, this process is influenced by CDK activity in mouse spermatocytes. At cap-remodeling the telomere ends are reorganized by the meiosis-specific TERB1/2-MAJIN connecting complex to ensure a stable telomere-NE attachment (Shibuya et al., 2015; Chen et al., 2021). Treatment with the CDK inhibitor Roscovitine was shown to avoid cap-remodeling. Interestingly, TERB1 Thr647 is a CDK susbtrate implicated in the downregulation of the TERB1-TRF1 interaction (Shibuya et al., 2014) (Figure 2). This phosphorylation was detected in NE-attached telomeres and shown to be important for the stabilization of telomere attachments (Shibuya et al., 2015). However, other CDK targets must exist to regulate cap-remodeling, since TERB1 phosphorylation is not essential for this process and the *TERB1-T647A* mutant is able to perform cap-remodeling. CDK2 could potentially exert this regulation given its essential function in telomere dynamics.

How the LINC and TERB1/2-MAJIN complexes interact to anchor telomeres to the NE was not clear, althought an interaction between SUN1 and TERB1 was reported (Shibuya et al., 2014). Recent studies dissecting these interactions, as well as the interaction and structural studies with Speedy A, support a model whereby Speedy A interacts with SUN1 to anchor CDK2-Speedy A to the NE which promotes SUN1 phosphorylation and the strengthening of the SUN1-MAJIN interaction. In addition, SUN1 also binds to TERB1 reinforcing the interaction between the complexes (Wang et al., 2020; Chen et al., 2021). Intestingly, in contrast to Speedy A, cyclin E1 and E2 do not bind to SUN1, pointing to Speedy A as the key triggering element to promote telomere-NE attachments (Wang et al., 2020). However, which SUN1 residues are phosphorylated and, in particular, the implication of Ser48 phosphorylation in MAJIN binding is unknown. Overall, CDK phosphorylation of SUN proteins seems to play an important role in the LINC-complex regulation; in fact, in worms and humans CDK1 is required for the phosphorylation of SUN proteins in mitosis (Patel et al., 2014; Zuela and Gruenbaum, 2016). Strikingly, in the case of worms, the expression in adults of the phosphomimetic SUN1-S34E protein drastically reduces fertility and abolishes bivalent formation (Zuela and Gruenbaum, 2016). Additionally, SUN1 phosphorylation mediated by other kinases controls meiotic chromosome dynamics in this organism (Penkner et al., 2009; Woglar et al., 2013).

*Bouquet* formation is regulated by a meiosis-specfic B3-type cyclin, Cyc2p, in *Tetrahymena thermophila*. In the absence of this cyclin micronuclei arrest at early prophase and fail to form the elongate-crecent shape characteristic of the *bouquet*-like organization in this ciliate (Xu et al., 2019). Cyc2p might be required to organize the microtubules that support crecent formation, which could explain the phenotype.

Finally, in addition to the positive effect of CDK phosphorylation in *bouquet* formation and telomeric dynamics, CDK regulation of *bouquet* disassembly has been reported in the budding yeast *Saccharomyces cerevisiae*. In this organism where *bouquet* conformation is very transient, controlled chemical inhibition of Cdc28 (CDK) stabilizes telomere clustering (Prasada Rao et al., 2021). However, the mechanism is currently unknown.

### DSB formation

A key feature of meiosis is recombination, the physical exchange of genetic information between each pair of parental chromosomes (homologs) (Hunter, 2015). In addition to produce variability in the offspring, it is essential for generating tension in the spindle-bound homologous pair which ensures proper alignment and chromosome segregation (Petronczki et al., 2003). Recombination is initiated by programmed DSBs in the DNA introduced by Spo11, a conserved meiosis-specific topo-like protein similar to TopVI of archaea (Keeney et al., 1997; Lam and Keeney, 2014; Bouuaert and Keeney, 2016; Robert et al., 2016; Vrielynck et al., 2016). DSBs are one of the most dangerous lesions in the DNA and cells have developed surveillance mechanisms to sense and repair these lesions. However, meiotic cells have integrated their production as part of the cell physiology, and have acquired a network of mechanisms to place and balance them (Keeney et al., 2014). DSB formation is coordinated with meiotic progression and DSBs occur locally after DNA replication during prophase (Borde et al., 2000; Murakami and Keeney, 2014). Indeed, replication-fork stalling blocks DSB formation by the activation of the S-phase checkpoint (Tonami et al., 2005; Ogino and Masai, 2006; Blitzblau and Hochwagen, 2013). In budding yeast, CDK (Cdc28) activity participates in this coordination phosphorylating Mer2, a conserved Spo11-accessory protein of the RMM complex (Henderson et al., 2006; Murakami and Keeney, 2008; Wan et al., 2008; Claeys Bouuaert et al., 2021). S-phase Cdc28 activity (associated to cyclin Clb5 and Clb6) phosphorylates Mer2 at Ser30 which primes adjacent Ser29 (and S28) for subsequent phosphorylation by DDK (Dbf4-Dependent Kinase). Mer2 phosphorylation at these sites is essential to promote the binding to other Spo11-accessory proteins, Spo11 loading at the recombination *hotspots* in the DNA, and DSB formation. However, additional CDK and DDK targets exist since the DSBs observed in cells expressing the phosphomimetic Mer2-DDD protein still depends on each of these kinases (Wan et al., 2008). Mer2 phosphorylation likely occurs upon replication-fork passage since DDK travels with the fork, and fork passage correlates with the loading onto chromatin of the Spo11-accessory protein Rec114, which it is known to depend on Mer2 phosphorylation (Panizza et al., 2011; Murakami and Keeney, 2014).

DSB formation also depends on CDK in fission yeast (Bustamante-Jaramillo et al., 2019). Although several CDK complexes contribute to this function, the meiosis-specific Crs1 cyclin has a prominent role and *crs1* mutants show a 50% reduction in DSB and recombination levels. In addition to its SPB location (see above), Crs1 has a pan-nuclear localization during meiotic prophase compatible with this role in DSB formation (Bustamante-Jaramillo et al., 2021). Interestingly, CDK downregulation impairs the chromatin binding of Rec25, a structural component of the Linear Elements (LinEs). LinEs are chromosome axis structures with similarity to the SC axial/lateral elements of other eukaryotes (Lorenz et al., 2004; Davis et al., 2008; Ding et al., 2021; Chuang and Smith, 2022). They are required for DSB formation and preferentially enriched at recombination *hotspots*; furthermore, when misplaced they induce DSBs (Fowler et al., 2013; Martin-Castellanos et al., 2013; Nambiar and Smith, 2018). Thus, modulation of the loading of LinEs onto chromatin may be a mechanism for CDK to control DSB formation. However, phosphonull *rec10-8A* or *rec27-A* mutants, the only two LinE-components harboring CDK sites, have no impact on meiotic recombination, even though several CDK sites in Rec10 are phosphorylated *in vivo* (Spirek et al., 2010; Bustamante-Jaramillo et al., 2019). Thus, it seems that these alterations alone would not significantly impair LinE chromatin association. In addition, similarly to budding yeast Mer2, the conserved RMM component Rec7 is a phosphoprotein that harbors a CDK site adjacent to potential DDK sites (Thr243 Ser244 Ser245) and DDK is also required for DSB formation (Ogino et al., 2006; Miyoshi et al., 2012). However, phosphonull *rec7-AAA* mutants show wild type levels of recombination (Bustamante-Jaramillo et al., 2019). Thus, a key target for this function of CDK has not yet been identified in fission yeast. It is possible that in this organism several CDK targets contribute to the regulation of DSB formation, and the reduction in recombination would not be observed until cumulative deregulation of several targets. Alternatively, regulation may be indirect. In this regard, LinE formation depends on the meiotic cohesins Rec8 and Rec11 which are phosphoproteins with several phospho-CDK sites detected *in vivo* (Molnar et al., 2003; Lorenz et al., 2004; Davis et al., 2008; Ishiguro et al., 2010; Rumpf et al., 2010; Fowler et al., 2013; Phadnis et al., 2015). Moreover, different levels of CDK regulation may exists. The cyclin Cig2-CDK complex regulates promoter occupancy of the mid-meiotic genes by phospho-regulation of the forkhead transcription factor Fkh2 (Alves-Rodrigues et al., 2016). Cig2-CDK phosphorylation of Fkh2 Ser481 reduces its promoter binding affinity and facilitates the loading of the transcriptional activator Mei4. One of these genes is *mde2*, which is essential for the organization of the pre-recombination complexes (Miyoshi et al., 2012). Therefore, timely expression of *mde2* by CDK might establish a temporal window for DSB formation.

REC-1 and HIM-5 are paralog related proteins required for normal levels of DSBs in *Caenorhabditis elegans* (Chung et al., 2015). Foci of the recombinase RAD-51 are reduced in each single mutant, particularly in *him-5*, and the reduction is significantly aggravated in the double mutants. This defect correlates well with the percentage of univalents at diakinesis (last prophase stage with highly condensed chromosomes). It was proposed that these proteins may function as Spo11-accessory proteins similarly to the proteins forming the pre-recombination complexes in budding and fission yeast; however, this has not been formally determined, and indeed, in contrast to Spo11-accessory proteins in yeasts, these *C. elegans* proteins are not essential for DSB formation. Interestingly, REC-1 is a CDK target that is phosphorylated *in vitro* by recombinant Cyclin B3-CDK4 complexes; moreover, it is also phosphorylated by cell extracts but not when the extracts are previously treated with Roscovitine (Chung et al., 2015). However, the *in vivo* relevance of this phosphorylation is unclear since both phosphomimetic and phosphonull *rec-1* mutants show a loss-of-function phenotype. It is possible that CDK does not play a crucial role in early meiotic events in this organism, where the meiosis-specific checkpoint kinase CHK-2 has acquired a prominent role in the regulation of chromosome architecture and movements, DSB formation, and homologous pairing and synapsis (MacQueen and Villeneuve, 2001; Penkner et al., 2009; Rosu et al., 2013; Stamper et al., 2013; Kim et al., 2015).

### Recombinational repair

**FIGURE 3:**
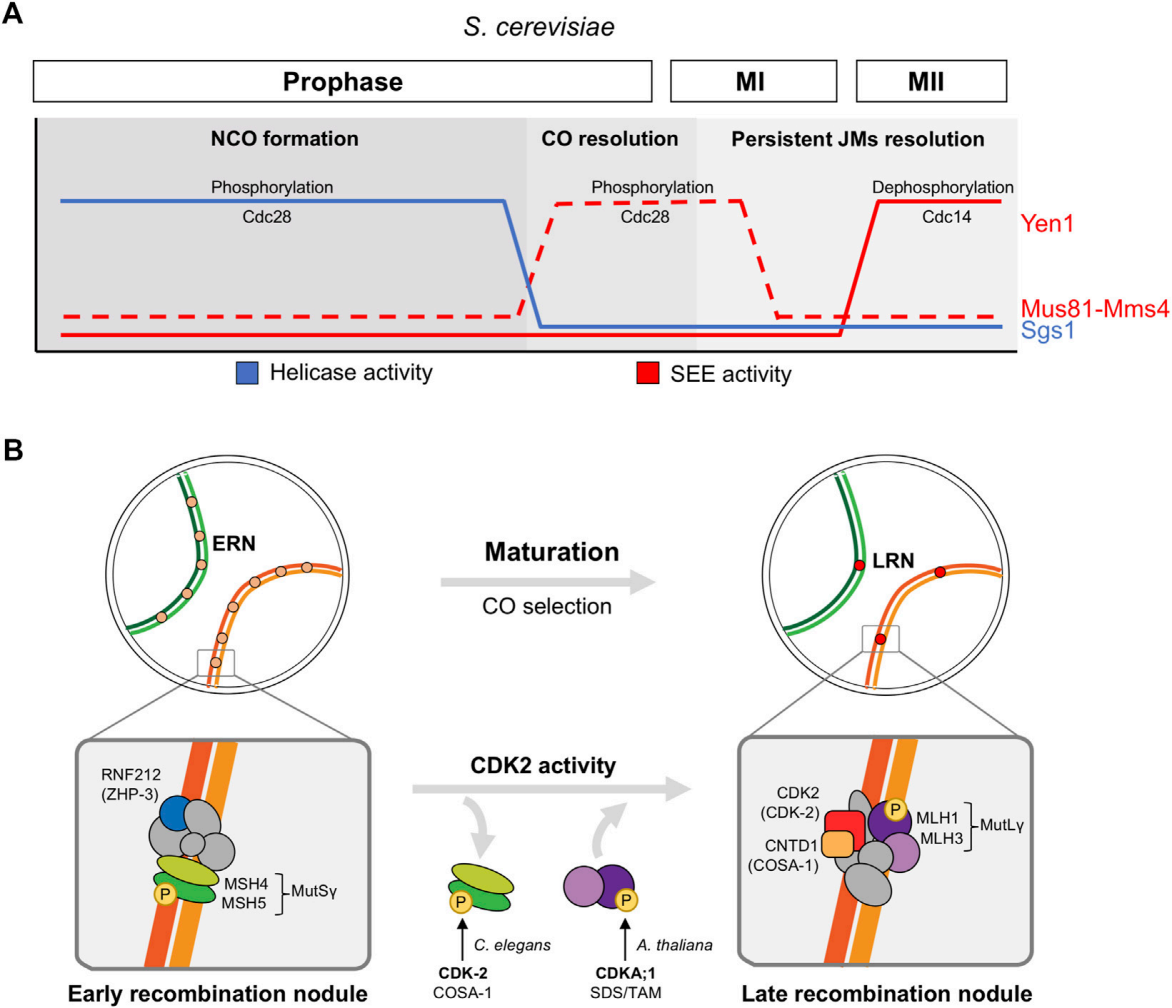
CDK regulation of recombinational repair in meiosis. **(A)** Role of CDK (Cdc28) in the sequential regulation of helicase and structure-selective endonuclease (SEE) activities in *S. cerevisiae*. Low Cdc28 activity activates the RecQ-family DNA helicase Sgs1 during early prophase to promote NCO formation, high levels of Cdc28 activity at meiosis I entry activates Mus81-Mms4 SSE to implement CO resolution prior to chromosome segregations, and Yen1 SSE activation by the Cdc14 phosphatase at meiosis II releases its earlier Cdc28-dependent inhibition to process persistent unresolved joint molecules (JMs). **(B)** Role of CDK2 in the maturation of early recombination nodules (ERN) and CO selection (late recombination nodules, LRN) in higher eukaryotes. Protein complexes are only illustrative and they do not represent protein-protein interaction nor stoichiometry. Only key proteins conserved in several organisms are highlighted: MutSγ complex MSH4-MSH5, RNF212 (ZHP-3 in *C. elegans*), MutLγ complex MLH1-MLH3, CDK2 (CDK-2 in *C. elegans*, CDKA;1 in *A. thaliana*), CNTD1 (COSA-1 in *C. elegans*). These proteins are key factors that reduce foci numbers from early pachytene to mid-pachytene (RNF212 and MutSγ) and that load at LRNs from mid pachytene (MutLγ, CDK2, and CNTD1).

After DSB formation Spo11-bound break sites are endonucleolytically resected to generate single-stranded DNA nucleofilaments that, coated with strand-exchange proteins Rad51/Dmc1, invade the homologous chromosome for repair. The efficient and differential resolution of the recombination intermediates is essential for several reasons (Hunter, 2015; San-Segundo and Clemente-Blanco, 2020). First, to produce viable gametes without DNA lesions. Second, to resolve enough DNA joint molecules (JM) as crossovers (CO). COs result in the reciprocal exchange of genetic material and the formation of physical links between the pair of homologs, which ensures their correct reductional segregation at first meiotic division (meiosis I). And finally, to resolve and release the physical connections between chromosomes on time before anaphase; otherwise, persistent JMs can impede chromosome segregations. Regarding this, some structure-selective endonucleases (SSEs) as Mus81-Mms4 and Yen1 in *S. cerevisiae* are tightly temporally controlled by cell cycle-regulated phosphorylation cycles to ensure that JMs are resolved and eliminated (Blanco and Matos, 2015) (Figure 3A). Of note that although CDK regulated targets that operate in DNA damage repair during vegetative growth has been broadly documented (reviewed in (Trovesi et al., 2013; Kciuk et al., 2022)), this is not the case for the meiotic DSB repair. It is expected that some of these targets are common to both mitotic and meiotic repair, while others will be specific to meiotic cells, given the formation of JMs between homologous chromosomes and the biased repair to generate COs.

In *S. cerevisiae* Mus81-Mms4 SSE activity is regulated by phosphorylation in meiotic and mitotic cells (Matos et al., 2011). Mms4 hyperphosphorylation in meiosis I strongly increases the nuclease activity of the complex, promoting the resolution of JMs before anaphase I. *mms4* deletion mutant accumulates aberrant multichromatid JMs. The non-phosphorylatable *mms4-14A* mutant shows a delay in the resolution of JMs and, similarly to *mus81* deletion mutants, fails to segregate homologous chromosomes at anaphase I (Matos et al., 2011). Despite the fact that Polo Kinase Cdc5 seems to play the major role in Mus81-Mms4 phospho-regulation, CDK (Cdc28) is involved as well, although this CDK regulation have mostly been studied in mitosis (Gallo-Fernandez et al., 2012; Matos et al., 2013; Szakal and Branzei, 2013). Indeed, the Mms-14A protein harbors changes to alanines in the 5 CDK sites present in the protein, and at least one of them is phosphorylated *in vivo*. The other ones were not confirmed due to incomplete peptide coverage of the mass spectrometry (MS) analysis (Matos et al., 2011). Cyclin Clb1-CDK activity is induced at the end of prophase (Carlile and Amon, 2008), and therefore, it is a candidate to participate in this regulation.

Mechanisms of CDK regulation have also been identified in fission yeast Mus81-Eme1 and human MUS81-EME1 in vegetative cells. In *S. pombe*, CDK (Cdc2)-dependent phosphorylation of Eme1 is cell-cycle regulated, and it is required to respond to DNA damage and to maintain chromosome stability in the absence of the RecQ-type DNA helicase Rqh1 (Dehe et al., 2013). In contrast to *S. cerevisiae*, fission yeast Eme1 phosphorylation does not depend on Polo kinase. In human cells, CDK stimulates MUS81-EME1 resolvase activity by promoting the interaction with SLX1-SLX4 SSE (Wyatt et al., 2013; Payliss et al., 2022). However, the relevance in meiosis of these phospho-regulations have not been studied.

Yen1 SSE has been widely studied in *S. cerevisiae*. During meiotic cell cycle, in sharp contrast to Mms4, CDK (Cdc28)-mediated phosphorylation of Yen1 restrains its function until anaphase II when it acts as an additional back-up system for late persistent JMs (Matos et al., 2011; Alonso-Ramos et al., 2021). *yen1* deletion mutant does not present obvious defects in chromosome segregations under unchallenged conditions, but additional deletion of other repair pathways as Sgs1 and Mus81-Mms4 results in defective JM resolution and abnormal anaphase I and II segregations, that unveils the safeguard activity of Yen1 (Matos et al., 2011; Alonso-Ramos et al., 2021). The specific mechanism of Yen1 dual phospho-regulation by Cdc28 and Cdc14 phosphatase was first elucidated in vegetative cells (Blanco et al., 2014; Eissler et al., 2014; Garcia-Luis et al., 2014), but it has also been described in meiosis. Cdc28-mediated phosphorylation not only inhibits Yen1 catalytic activity, but also prevents its precocious nuclear accumulation. At anaphase II, Cdc14 released from the nucleolus dephosphorylates Yen1, promoting its activation and nuclear enrichment (Alonso-Ramos et al., 2021). Regarding this mechanism, a mutant protein where the 9 CDK-consensus serines are mutated to alanines, named Yen1^ON^, bypasses this phospho-regulation. Therefore, Yen^ON^ is constitutively active and accumulated in the nucleus, even in early stages of meiosis (as S-phase and prophase), in a Cdc14 independent manner (Blanco et al., 2014; Arter et al., 2018; Alonso-Ramos et al., 2021). Early Yen1 activation induces premature CO formation and avoids the transitory accumulation of JMs. As a consequence, some aspects of the CO physiology such as CO interference (nearby CO inhibition) and distribution are defective (Arter et al., 2018). Although Yen1 reaches its maximum activity in meiosis II, some evidence indicates that it might exert a previous function at anaphase I, especially when other repair pathways are compromised (Alonso-Ramos et al., 2021).

Similarly to Yen1, human ortolog GEN1 contains several CDK consensus sites, although they are not conserved in position and context. Indeed, GEN1 is phosphorylated in a CDK-dependent manner in mitosis; however, this modification has no impact on the catalytic activity, and wild type and GEN1-8A proteins resolve JMs with the same efficiency (Chan and West, 2014). Furthermore, the relevance of this modification in meiosis has not been studied.

The same phospho-regulatory network that tightly controls SSE activity during nuclear divisions, also regulates Sgs1 in *S. cerevisiae* meiosis and mitosis (Grigaitis et al., 2020) (Figure 3A). The RecQ-family DNA helicase Sgs1, together with Top3 and Rmi1 (STR complex), contributes to DSB repair by disassembling different recombination intermediates favouring the production of non-crossover (NCO) products over COs. In addition, a meiotic pro-CO function of Sgs1 is uncovered in the absence of SSEs (Zakharyevich et al., 2012). During meiotic S-phase and prophase, CDK (Cdc28) phosphorylation of Sgs1 substantially increases its DNA unwinding activity. This phospho-stimulation is essential for proper JM processing, and the phosphonull *sgs1-9A* mutant accumulates aberrant multichromatid JMs. Indeed, *sgs1-9A* mutant fails to segregate homologous chromosomes at anaphase I, a defect that is enhanced in the absence of Mms4 and alleviated in the presence of the Yen1^ON^ version. Cdc28 phosphorylation also primes Sgs1 for Cdc5 hyperphosphorylation as cells exit prophase, what has been suggested to inhibit its activity, but the meaning of this modification is not completely elucidated (Grigaitis et al., 2020). It is tempting to think that the differential phosphorylation state may modulate Sgs1 functions to promote NCOs in early prophase and JM resolution later on prior to chromosome segregation as SSEs do.

Overall, CDK plays a crucial role in the metabolism of the JMs generated during meiotic DSB repair, at least in budding yeast (Figure 3A). It enhances or inhibits different activities involved in the process and, in doing so, it temporally orders their actions to ensure an efficient JM processing that guarantees a faithful chromosome segregation. In addition, it restrains JM resolution to stablish a correct CO pattern.

It is quite possible that the role of CDK in averting late JMs is a conserved feature. In fission yeast, the UvrD-type DNA helicase Fbh1 is required to remove Rad51 from the DNA. Both, *fbh1* mutants, and mutants in its loading factor *dbl2*, show a retention of Rad51 foci at meiosis I and defects in chromosome segregation (Sun et al., 2011; Polakova et al., 2016); moreover, persistence of JMs is observed in the *dbl2* mutant (Polakova et al., 2016). Interestingly, Dbl2 is potentially a good CDK substrate. It harbours several putative CDK sites, and 3 of them are phosphorylated in vegetative cycle (https://www.pombase.org/gene/SPCC553.01c). It will be worthy to explore any meiotic contribution of CDK to Dbl2 function and JM resolution.

In fission yeast the meiosis-specific Rem1 cyclin is required for normal levels of recombination. Interestingly, in its absence NCOs are reduced but CO levels are not affected, suggesting a regulatory role in DSB repair (Malapeira et al., 2005). However, this function does not depend on CDK (Cdc2). Intron retention produces a short isoform that lacks the cyclin-box motif involved in Cdc2 binding, and this short version restores the recombination defects of the *rem1* mutant (Moldon et al., 2008). It is currently unknown how short Rem1 controls the recombination output. Intron processing depends on Mei4 binding to *rem1* promoter, which recruits the spliceosome, and produces a larger protein that shows a peak of associated kinase activity at meiosis I and promotes meiosis I progression (Malapeira et al., 2005; Moldon et al., 2008). Thus, Rem1 represents an example of gene economy where splicing regulation produces a cyclin and a non-cyclin protein with different meiotic functions. Differential gene expression regulation has been also reported for other meiotic genes. For the unconventional CNTD1 mouse cyclin a short isoform has been recently described as the only detectable species in mouse testis (see below, (Gray et al., 2020)). In this case, the function of the full length CNTD1 protein is unclear. Interestingly, mouse CDK2 also shows a larger isoform *via* alternative splicing that is highly induced in meiotic prophase (Ellenrieder et al., 2001; Liu et al., 2014; Tu et al., 2017). It has been proposed that the larger protein may add new binding domains to accommodate different meiotic functions. Indeed, some interactors preferentially bind to the long CDK2 isoform *in vitro* (Liu et al., 2014; Bondarieva et al., 2020).

In mouse, in addition to its telomeric localization, CDK2 is also observed in 1-2 interstitial sites along the chromosomal axes in mid-pachytene spermatocytes and oocytes (Ashley et al., 2001) (Figure 3B). At these spots, it colocalizes with late recombination nodule (LRN) pro-CO factors as RNF212, HEI10, PRR19, CNTD1, and MLH1, and this localization is lost in the corresponding mutants (Ashley et al., 2001; Ward et al., 2007; Reynolds et al., 2013; Holloway et al., 2014; Liu et al., 2014; Qiao et al., 2014; Bondarieva et al., 2020). Based on localization studies, CDK2 was proposed to act with the HEI10 E3-sumo-targeting ubiquitin ligase in the dissociation of early recombination factors and final CO selection (Qiao et al., 2014). Interestingly, human HEI10 was shown to interact with cyclin B1 in Y2H assays, and it is specifically phosphorylated *in vitro* by cyclin B-Cdk1 complexes (Toby et al., 2003); however, cyclin B1-Cdk1 has not been implicated in CO selection. Recently, the use of a partial loss-of-function and a gain-of-function *Cdk2* allele has helped to further explore the role in CO regulation of CDK2 activity (Palmer et al., 2020). The hypomorphic *Cdk2* ^*T160A*^ allele harbors a point mutation in the so-called T-loop of the protein. Upon binding to the cyclin, the loop is displayed out of the catalytic cleft and exposed to phosphorylation by CAK (CDK activating kinase), stabilizing the complex and, therefore, promoting maximal activation (Malumbres, 2014). Telomeric CDK2 localization and function remains mostly unaffected in *Cdk2* ^*T160A*^ mutants, which allows the study of CDK2 roles in later events (Palmer et al., 2020). CDK2 location in LRNs depends on T-loop phosphorylation since *Cdk2* ^*T160A*^ spermatocytes lose these interstitial foci and the associated MLH1 loading (component of the conserved MutLγ SSE with bias for CO resolution). Correspondingly, increased kinase activity in the gain-of-function *Cdk2* ^*Y15S*^ mutant, which precludes inhibition by phosphorylation at Tyr15 in the catalytic pocket (Malumbres, 2014), correlates with elevated numbers of interstitial foci and the associated MLH1 loading. In addition, similarly to other mutants in pro-CO factors, *Cdk2* ^*T160A*^ spermatocytes show persistence of foci of earlier recombination proteins as RPA2 and MSH4 (component of the conserved MutSγ complex), reflecting aberrant stabilization and repair of intermediates. In particular, the typical reduction in RNF212 foci correlated with “selection” of meiotic CO sites does not occur in *Cdk2* ^*T160A*^ spermatocytes what indicates a defective CO designation process when CDK2 activity is reduced (Reynolds et al., 2013; Qiao et al., 2014; Palmer et al., 2020). Thus, CDK2 might phosphorylate substrates in the recombination nodules leading the repair process towards CO formation; however, the molecular mechanism is currently unknown. Interestingly, *Cdk2* ^*T160A*^ mutant primarily affects the activity of CDK2 complexes with conventional cyclin activators, since CDK2-Speedy A complexes do not require Thr160 phosphorylation for activation (Karaiskou et al., 2001). This excludes Speedy A from any role in this LRN associated function of CDK2; and, indeed, Speedy A is not located in interstitial foci along chromosomes axes (Mikolcevic et al., 2016; Tu et al., 2017). Furthermore, CDK2 phosphorylation at Thr160 is observed in LRNs and not in telomeres (Liu et al., 2014).

The role of CDK2 in the maturation of early recombination sites and CO designation is conserved in *C. elegans* (Figure 3B). CDK-2 homolog also localizes at CO-selected sites, and it is eventually detected as six strong foci in pachytene (one per homolog pair). Absence of bivalents in CDK-2 depleted oocytes reflects a failure to properly generate COs. Specifically, the CO selection pathway is disrupted, as seen by the fact that ZHP-3 (RNF212 in mouse) and MSH-5 (component of the conserved MutSγ complex) signals fail to restrict to the six CO sites as normally happens in late pachytene (Haversat et al., 2022). Some studies identified the cyclin-like protein COSA-1 (Crossover Site-Associated-1) as a partner for CDK-2 in *C. elegans* meiosis. COSA-1 and CDK-2 colocalize at CO sites in a mutually-dependent manner and their absence similarly impairs the dynamics of CO designation factors, what support their functional association (Yokoo et al., 2012; Haversat et al., 2022). In the *cosa-1* mutant, bivalents are also absent, and at late pachytene ZHP-3 aberrantly remains at high levels along the SC and MSH5 foci are lost (Yokoo et al., 2012). Moreover, *in vitro* experiments showed that CDK-2 and COSA-1 are able to form a complex (Haversat et al., 2022).

Key meiotic substrates of CDK2 for this crossover-related function are not clearly determined. Recent studies describe MSH-5 as a key target in *C. elegans* (Haversat et al., 2022). MSH-5 contains thirteen CDK consensus motifs in a disordered C-terminal tail, and this domain is essential for its function in CO formation. In a C-terminal truncated mutant (*msh-5* ^*∆339aa*^), ZHP-3 signal aberrantly persists and most recombination intermediates fail to mature into COs. Three of these sites are, indeed, *in vitro* phosphorylated by recombinant human CDK1-cyclin A2 complexes. Moreover, phospho-specific antibodies to one of these sites, Thr1009, show that MSH-5 is actually phosphorylated *in vivo* in a CDK-2 and COSA-1 dependent manner. Phosphorylated-MSH5 signal was enriched at CO-designated sites in late pachytene, colocalizing with COSA-1 during meiotic progression. CDK phosphorylation of MSH-5 promotes its pro-CO activity since, in a sensitized condition (*him-14(it44)* mutant, MSH4 in mouse), the *msh5* ^*13A*^ phospho-null mutant is unable to form bivalents. Moreover, at late prophase colocalization between MSH-5^13A^ and COSA-1 is lost and the MSH-5^13A^ protein persists as multiple foci instead of pairing down to six bright foci, reminiscent of what it is observed in CDK-2 depleted germlines. The disordered C-terminal tail containing CDK sites in MSH-5 is not conserved outside *Caenorhabditis* species, indicating that, although the role of CDK2 in CO selection is conserved, the mechanism may differ from one organism to another (Haversat et al., 2022).

The mammalian COSA-1 homolog, CNTD1, also presents a conserved function in CO selection (Figure 3B). It is highly enriched in mouse and human testis, and in mouse spermatocytes CNTD1 foci are observed at CO sites colocalizing with CDK2 and MLH1 in mid-pachytene (Bondarieva et al., 2020; Gray et al., 2020). Similarly to other CO machinery mutants (as *Mlh1*, *Mlh3*, *Hei10*, *Rnf212*), when Cntd1 function is depleted early prophase events including homolog pairing and initial DSB processing remain normal, but severe defects in DSB repair and CO formation are observed (Holloway et al., 2014). In *Cntd1* mutant spermatocytes, MutLγ complex (MLH1 and MLH3) and CDK2 do not localize to LRNs, indicating a disruption in the canonical CO pathway. In addition, the earlier recombination factors MSH4 and RNF212 are not properly removed from these sites as seen by persistence (or even increase) of foci and co-foci of these proteins during late pachytene. Thus, CNTD1 seems to act specifically in the final selection of CO sites, coordinating RNF212 and MutSγ dissociation with the recruitment of MutLγ.

The identification of CNTD1 as a member of the cyclin superfamily indicates that it may function in a CDK-CNTD1 kinase complex. CDK2 is a good partner for CNTD1 because its associated kinase activity is also involved in CO designation (see above); in fact, interstitial CDK2 foci are absent in *Cntd1* mutants and, although the interaction has not been detected *in vivo*, CNTD1 has been shown to interact with CDK2 by Y2H assays (Holloway et al., 2014; Bondarieva et al., 2020; Gray et al., 2020). The CNTD1–CDK2 interaction requires the first predicted cyclin box located in CNTD1 N-terminus; hence, mutants that alter this first cyclin box diminish the interaction in Y2H assays and impair CNTD1 function (Bondarieva et al., 2020). Consistent with this mechanism, other studies have identified a short CNTD1 isoform that lacks the first cyclin homology domain and is not able to interact with CDK2 by Y2H assays (Gray et al., 2020). Currently, the function of the long CNTD1 protein is unclear, since apparently the short form is the only detectable species in adult mouse testis (Gray et al., 2020). Further studies are needed to shed light into the function and regulation of the distinct CNTD1 isoforms. Given that long variants have been found in different vertebrates including mouse (Gray et al., 2020), the use of specific antibodies for the detection of the long isoforms will be particularly informative. Nevertheless, given that CDK2 activity is required for CO selection, there must be other cyclin/s or activators/s to provide this activity.

Molecularly, in extracts from adult testis no relevant interactions between short-CNTD1 and the CO machinery (MSH4, MSH5, MLH1, MLH3, RNF212, HEI10, or CDK2) have been identified by MS (Gray et al., 2020). However, MS data shows unexpected interactions with components of the Replication Factor C (RFC) complex, the loader of the Proliferating Cell Nuclear Antigen (PCNA). Indeed, RFC3, RFC4 and PCNA proteins are expressed during meiotic prophase (after DNA replication), and in the case of RFC4 clearly detected forming CNTD1-dependent foci on synapsed chromosomes in pachytene spermatocytes. Given the role of human RFC and PCNA in the activation of the MutLγ endonuclease complex *in vitro*, and the localization of PCNA at a subset of prospective CO sites in budding yeast cells arrested in pachytene (Cannavo et al., 2020; Kulkarni et al., 2020), it has been proposed that CNTD1 association with components of the RFC-PCNA complex would stimulate MutLγ activity and promote CO formation (Gray et al., 2020).

In *Arabidopsis thaliana* CDK activity also regulates CO formation (Figure 3B). CDKA;1, the homolog for mammalian CDK1 and CDK2, regulates CO formation in a dose-dependent manner (Wijnker et al., 2019). Though CDKA;1 is essential, weak loss-of-function alleles are viable and produce flowers containing abnormal meiocytes (Dissmeyer et al., 2007; Wijnker et al., 2019). The hypomorphic *cdka;1* ^*DBD*^ mutant expresses a fusion of *CDKA;1* to an inactive (dead) destruction box of *CYCLINB1;1*, and it exhibits partial kinase activity and reduced fertility (Wijnker et al., 2019). Early stages of prophase are largely unaffected in *cdka;1* ^*DBD*^ mutants; however, at diplotene reduced numbers of bivalents are often observed, and univalents are also frequent at metaphase I. The conventional CO pathway is affected as seen by a significant decrease of approx. 50% in MLH1 foci. Conversely, enhanced CDKA;1 activity increases recombination by approx. 10%. Regarding the molecular mechanism, *in vitro* kinase assays show that CDKA;1 in complex with meiosis-specific cyclins SDS (SOLO DANCERS) or TAM (TARDY ASYNCHRONOUS MEIOSIS) can phosphorylate MLH1, especially the CDKA;1-SDS complex, what can represent a mechanism to control CO formation. Interestingly, the SDS cyclin contains an unusually long N-terminal region. This structure resembles COSA-1/CTND1 unconventional cyclins, whose sequences also comprise an insertion of 26–33 amino acids in the highly conserved N-terminal cyclin box domain (Azumi et al., 2002; Yokoo et al., 2012).

### Synapsis

The synapsis of homologous chromosomes is essential for recombination given the close proximity required for recombination to occur. This physical constraint has evolutionarily associated both processes and recombination occurs in the context of the SC; moreover, in many organisms as budding yeast, mouse, and plants, early recombination intermediates promote SC formation and chromosome synapsis (Zickler and Kleckner, 2015). However, CDK plays a role in SC regulation independently of its function in DSB formation, and some SC proteins are known CDK substrates.

In budding yeast, CDK (Cdc28) foci appear early in prophase and depend on S-phase Clb5 and Clb6 cyclins and the axial-element components Red1 and Hop1 (Zhu et al., 2010). Later on, Cdc28 tends to localize on synapsed chromosomes. Chemical inhibition of Cdc28 after DSB formation impairs polymerization of the SC central-element component Zip1. However, although Zip1 is an *in vitro* CDK substrate (Ubersax et al., 2003) and its electrophoretic gel-mobility depends on Cdc28, Clb5, and Clb6, a phospho-null Zip1-4SA protein supports normal SC formation (Zhu et al., 2010). Additionally, Red1 is also phosphorylated in a Cdc28-depedent manner independently of DSB formation (Lai et al., 2011). Even though the phospho-null Red1-7A protein is hypophosphorylated during meiotic prophase, it does not impair sporulation efficiency or spore viability, suggesting normal SC formation (Lai et al., 2011). Thus, although SC formation requires CDK activity in budding yeast, it is not well understood how this function is accomplished. It is possible that multiple CDK regulated pathways converge to ensure efficient SC formation. Moreover, given the link between recombination and SC formation in this organism, it is possible that a putative CDK role downstream of DSB formation may also contribute to SC development.

Similarly, CDKA;1 is required for ZYP1 (Zip1) assembly in *Arabidopsis* meiotic chromosomes (Yang et al., 2020). The infertile hypomorphic *cdka;1* ^*T161D*^ allele harbors a point mutation in the so-called T-loop of the protein that impairs fully activation (see above) (Dissmeyer et al., 2007). *cdka;1* ^*T161D*^ mutants show pachytene-like meiocytes with ZYP1-depleted unpaired chromosomes and the absence of bivalents. The mutant does not affect Dmc1 recombinase loading, indicating that the synaptic defect is not due to the lack of DSBs. CDKA;1 co-localizes with ASY1 (Hop1 homolog) at chromosomes axis and both proteins disappear from the axis of synapsed chromosomes. ASY1 is a CDKA;1 substrate and, particularly, phosphorylation of Thr142 and Thr184 residues in the HORMA domain of the protein promotes self-interaction and increases the binding affinity to ASY3 (Red1 homolog), which facilitates ASY1 chromosomal loading (Yang et al., 2020).

CDKG is also required for full ZYP1 assembly in *Arabidopsis* (Zheng et al., 2014). In this case, the function is sex and environmental-condition specific, since the null *cdkg1-1* mutant exhibits only male sterility under high temperature. Regarding the mechanism, an indirect role through gene expression regulation was proposed, since CDKG associates with the spliceosome and controls the expression of a gene involved in pollen differentiation (Huang et al., 2013). The conserved role of CDKs in mRNA metabolism is well documented (Malumbres, 2014).

In mouse, the use of the hypomorphic *Cdk2* ^*T160A*^ allele (see above) has also uncovered a role in synapsis maintenance (Palmer et al., 2020). Since CDK2 is required for the telomere attachment to the NE early in prophase (see above), the observed defects in synapsis of the null mutant were difficult to evaluate as a direct consequence. The CDK2^T160A^ mutant protein localizes properly at telomeres, and spermatocytes progress normally to early pachytene with normal synapsed chromosomes. However, later on partially unsynapsed chromosomes are observed, and loading of the transverse element SYCP1 is diminished; by diplotene a complete separation of the homologs is frequently observed. As in budding yeast, mouse SC formation also depends on early recombination intermediates, and, as mentioned above, *Cdk2* ^*T160A*^ mutants accumulate RPA2, RNF212, and MSH4 foci, suggesting a problem in the processing of intermediates that might affect the stability of the SC (Palmer et al., 2020). However, since mutations in pro-CO factors also show an increased number of these foci but normal chromosome synapsis (Ward et al., 2007; Reynolds et al., 2013; Holloway et al., 2014; Bondarieva et al., 2020), CDK2 activity is likely to be directly involved in synapsis maintenance. Finally, given that *Cdk2* ^*T160A*^ primarily affects the activity of CDK2 complexes with conventional cyclin activators (Karaiskou et al., 2001), this role in synapsis stabilization might depend on E cyclins (see above). The importance of CDK2 in different aspects of meiotic prophase, herein reviewed, has pointed it as a target for nonhormonal male contraception (Faber et al., 2020).

SC disassembly at the end of prophase is equally as important as SC formation. In *C. elegans* CDK-1 regulates the chromosomal relocation of the Polo kinase PLK-2 from the pairing centers to the SC at late pachytene (Brandt et al., 2020). SYP-1 (Zip1) is phosphorylated by CDK-1 at Thr452 located in the PBD (Polo Box Domain)-binding motif. This modification primes for PLK-2 binding that sustains SYP-1 phosphorylation and promotes SC disassembly. Meiotic depletion of CDK-1 does not affect PLK-2 recruitment to the pairing centers, and both synapsis and CO formation are normal. However, SYP-1-T452 phosphorylation is completely abolished and PLK-2 is not recruited to SC; as a consequence, chromosome axis remodeling is impaired, and SC disassembly is delayed until diakinesis (Brandt et al., 2020).

## Concluding remarks

Since the molecular identification of the MPF (Maturation Promoting Factor) activity of *Xenopus laevis* oocytes in the late 80′s, the study of cyclins and CDKs has generated a vast amount of knowledge on how eukaryotic cells divide and coordinate division with internal and external cues in many species. Indeed, cyclins and CDKs have emerged as broadly conserved key regulators of the eukaryotic cell cycle. However, how these regulators control important aspects of meiosis, particularly during prophase where critical cellular events for the generation of healthy gametes occur, is not well understood. In fact, as summarized in this review, no many direct CDK meiotic-targets have been clearly identified. In addition, meiosis represents a very interesting scenario to study CDK activity diversification with the emergence of meiosis-specific cyclins and new associated functions. The important role of CDK activity in meiosis is pointed by the fact that in a recent phosphoproteomic study of mouse spermatocytes undergoing prophase, 10 of the top 30 enriched kinases were CDKs with CDK2 in the second position (Li et al., 2022). The increased number of proteomic studies in several organisms, the development of improved techniques to enrich cell populations in specific prophase stages, along with the analysis of the corresponding phosphomutants, will help to broad our current knowledge of the fascinating process of gamete generation and the efficient genome transmission from one generation to the next.

## References

[B1] Alonso-Ramos P. Alvarez-Melo D. Strouhalova K. Pascual-Silva C. Garside G. B. Arter M. (2021). The Cdc14 phosphatase controls resolution of recombination intermediates and crossover formation during meiosis. Int. J. Mol. Sci. 22 (18), 9811. 10.3390/ijms22189811 34575966 PMC8470964

[B2] Alves-Rodrigues I. Ferreira P. G. Moldon A. Vivancos A. P. Hidalgo E. Guigo R. (2016). Spatiotemporal control of forkhead binding to DNA regulates the meiotic gene expression program. Cell Rep. 14 (4), 885–895. 10.1016/j.celrep.2015.12.074 26804917

[B3] Amelina H. Subramaniam S. Moiseeva V. Armstrong C. A. Pearson S. R. Tomita K. (2015). Telomere protein Rap1 is a charge resistant scaffolding protein in chromosomal bouquet formation. BMC Biol. 13, 37. 10.1186/s12915-015-0149-x 26058898 PMC4660835

[B4] Arter M. Hurtado-Nieves V. Oke A. Zhuge T. Wettstein R. Fung J. C. (2018). Regulated crossing-over requires inactivation of yen1/GEN1 resolvase during meiotic prophase I. Dev. Cell 45 (6), 785–800. e786. 10.1016/j.devcel.2018.05.020 29920281 PMC6043783

[B5] Ashley T. Walpita D. de Rooij D. G. (2001). Localization of two mammalian cyclin dependent kinases during mammalian meiosis. J. Cell Sci. 114 (4), 685–693. 10.1242/jcs.114.4.685 11171374

[B6] Azumi Y. Liu D. Zhao D. Li W. Wang G. Hu Y. (2002). Homolog interaction during meiotic prophase I in Arabidopsis requires the SOLO DANCERS gene encoding a novel cyclin-like protein. EMBO J. 21 (12), 3081–3095. 10.1093/emboj/cdf285 12065421 PMC126045

[B7] Bass H. W. (2003). Telomere dynamics unique to meiotic prophase: Formation and significance of the bouquet. Cell. Mol. Life Sci. 60 (11), 2319–2324. 10.1007/s00018-003-3312-4 14625678 PMC11138934

[B8] Berthet C. Aleem E. Coppola V. Tessarollo L. Kaldis P. (2003). Cdk2 knockout mice are viable. Curr. Biol. 13 (20), 1775–1785. 10.1016/j.cub.2003.09.024 14561402

[B9] Blanco M. G. Matos J. (2015). Hold your horSSEs: Controlling structure-selective endonucleases MUS81 and yen1/GEN1. Front. Genet. 6, 253. 10.3389/fgene.2015.00253 26284109 PMC4519697

[B10] Blanco M. G. Matos J. West S. C. (2014). Dual control of Yen1 nuclease activity and cellular localization by Cdk and Cdc14 prevents genome instability. Mol. Cell 54 (1), 94–106. 10.1016/j.molcel.2014.02.011 24631285 PMC3988869

[B11] Blitzblau H. G. Hochwagen A. (2013). ATR/Mec1 prevents lethal meiotic recombination initiation on partially replicated chromosomes in budding yeast. Elife 2, e00844. 10.7554/eLife.00844 24137535 PMC3787542

[B12] Bondarieva A. Raveendran K. Telychko V. Rao H. Ravindranathan R. Zorzompokou C. (2020). Proline-rich protein PRR19 functions with cyclin-like CNTD1 to promote meiotic crossing over in mouse. Nat. Commun. 11 (1), 3101. 10.1038/s41467-020-16885-3 32555348 PMC7303132

[B13] Borde V. Goldman A. S. Lichten M. (2000). Direct coupling between meiotic DNA replication and recombination initiation. Science 290 (5492), 806–809. 10.1126/science.290.5492.806 11052944

[B14] Bouuaert C. C. Keeney S. (2016). DNA. Breaking DNA. Science 351 (6276), 916–917. 10.1126/science.aaf2509 26917753

[B15] Brandt J. N. Hussey K. A. Kim Y. (2020). Spatial and temporal control of targeting Polo-like kinase during meiotic prophase. J. Cell Biol. 219 (11), e202006094. 10.1083/jcb.202006094 32997737 PMC7594494

[B16] Bustamante-Jaramillo L. F. Ramos C. Alonso L. Sesmero A. Segurado M. Martin-Castellanos C. (2019). CDK contribution to DSB formation and recombination in fission yeast meiosis. PLoS Genet. 15 (1), e1007876. 10.1371/journal.pgen.1007876 30640914 PMC6331086

[B17] Bustamante-Jaramillo L. F. Ramos C. Martin-Castellanos C. (2021). The meiosis-specific Crs1 cyclin is required for efficient S-phase progression and stable nuclear architecture. Int. J. Mol. Sci. 22 (11), 5483. 10.3390/ijms22115483 34067465 PMC8196990

[B18] Cannavo E. Sanchez A. Anand R. Ranjha L. Hugener J. Adam C. (2020). Regulation of the MLH1-MLH3 endonuclease in meiosis. Nature 586 (7830), 618–622. 10.1038/s41586-020-2592-2 32814904

[B19] Carlile T. M. Amon A. (2008). Meiosis I is established through division-specific translational control of a cyclin. Cell 133 (2), 280–291. 10.1016/j.cell.2008.02.032 18423199 PMC2396536

[B20] Chan Y. W. West S. C. (2014). Spatial control of the GEN1 Holliday junction resolvase ensures genome stability. Nat. Commun. 5, 4844. 10.1038/ncomms5844 25209024 PMC4172962

[B21] Chen Y. Wang Y. Chen J. Zuo W. Fan Y. Huang S. (2021). The SUN1-SPDYA interaction plays an essential role in meiosis prophase I. Nat. Commun. 12 (1), 3176. 10.1038/s41467-021-23550-w 34039995 PMC8155084

[B22] Chikashige Y. Tsutsumi C. Yamane M. Okamasa K. Haraguchi T. Hiraoka Y. (2006). Meiotic proteins bqt1 and bqt2 tether telomeres to form the bouquet arrangement of chromosomes. Cell 125 (1), 59–69. 10.1016/j.cell.2006.01.048 16615890

[B23] Chikashige Y. Yamane M. Okamasa K. Tsutsumi C. Kojidani T. Sato M. (2009). Membrane proteins Bqt3 and -4 anchor telomeres to the nuclear envelope to ensure chromosomal bouquet formation. J. Cell Biol. 187 (3), 413–427. 10.1083/jcb.200902122 19948484 PMC2779253

[B24] Chotiner J. Y. Wolgemuth D. J. Wang P. J. (2019). Functions of cyclins and CDKs in mammalian gametogenesis. Biol. Reprod. 101 (3), 591–601. 10.1093/biolre/ioz070 31078132 PMC6791058

[B25] Chuang Y. C. Smith G. R. (2022). Dynamic configurations of meiotic DNA-break hotspot determinant proteins. J. Cell Sci. 135 (3), jcs259061. 10.1242/jcs.259061 35028663 PMC8918816

[B26] Chung G. Rose A. M. Petalcorin M. I. Martin J. S. Kessler Z. Sanchez-Pulido L. (2015). REC-1 and HIM-5 distribute meiotic crossovers and function redundantly in meiotic double-strand break formation in *Caenorhabditis elegans* . Genes Dev. 29 (18), 1969–1979. 10.1101/gad.266056.115 26385965 PMC4579353

[B27] Claeys Bouuaert C. Pu S. Wang J. Oger C. Daccache D. Xie W. (2021). DNA-driven condensation assembles the meiotic DNA break machinery. Nature 592 (7852), 144–149. 10.1038/s41586-021-03374-w 33731927 PMC8016751

[B28] Cohen P. E. Pollard J. W. (2001). Regulation of meiotic recombination and prophase I progression in mammals. Bioessays 23 (11), 996–1009. 10.1002/bies.1145 11746216

[B29] Coudreuse D. Nurse P. (2010). Driving the cell cycle with a minimal CDK control network. Nature 468 (7327), 1074–1079. 10.1038/nature09543 21179163

[B30] Davis L. Rozalen A. E. Moreno S. Smith G. R. Martin-Castellanos C. (2008). Rec25 and Rec27, novel linear-element components, link cohesin to meiotic DNA breakage and recombination. Curr. Biol. 18 (11), 849–854. 10.1016/j.cub.2008.05.025 18514516 PMC3119532

[B31] Dehe P. M. Coulon S. Scaglione S. Shanahan P. Takedachi A. Wohlschlegel J. A. (2013). Regulation of Mus81-Eme1 Holliday junction resolvase in response to DNA damage. Nat. Struct. Mol. Biol. 20 (5), 598–603. 10.1038/nsmb.2550 23584455 PMC3978046

[B32] Ding D. Q. Matsuda A. Okamasa K. Hiraoka Y. (2021). Linear elements are stable structures along the chromosome axis in fission yeast meiosis. Chromosoma 130 (2-3), 149–162. 10.1007/s00412-021-00757-w 33825974 PMC8426239

[B33] Ding D. Q. Yamamoto A. Haraguchi T. Hiraoka Y. (2004). Dynamics of homologous chromosome pairing during meiotic prophase in fission yeast. Dev. Cell 6 (3), 329–341. 10.1016/s1534-5807(04)00059-0 15030757

[B34] Ding X. Xu R. Yu J. Xu T. Zhuang Y. Han M. (2007). SUN1 is required for telomere attachment to nuclear envelope and gametogenesis in mice. Dev. Cell 12 (6), 863–872. 10.1016/j.devcel.2007.03.018 17543860

[B35] Dissmeyer N. Nowack M. K. Pusch S. Stals H. Inze D. Grini P. E. (2007). T-loop phosphorylation of Arabidopsis CDKA;1 is required for its function and can be partially substituted by an aspartate residue. Plant Cell 19 (3), 972–985. 10.1105/tpc.107.050401 17369369 PMC1867360

[B36] Eissler C. L. Mazon G. Powers B. L. Savinov S. N. Symington L. S. Hall M. C. (2014). The Cdk/cDc14 module controls activation of the Yen1 holliday junction resolvase to promote genome stability. Mol. Cell 54 (1), 80–93. 10.1016/j.molcel.2014.02.012 24631283 PMC3988236

[B37] Ellenrieder C. Bartosch B. Lee G. Y. Murphy M. Sweeney C. Hergersberg M. (2001). The long form of CDK2 arises via alternative splicing and forms an active protein kinase with cyclins A and E. DNA Cell Biol. 20 (7), 413–423. 10.1089/104454901750361479 11506705

[B38] Faber E. B. Wang N. Georg G. I. (2020). Review of rationale and progress toward targeting cyclin-dependent kinase 2 (CDK2) for male contraception. Biol. Reprod. 103 (2), 357–367. 10.1093/biolre/ioaa107 32543655 PMC7523694

[B39] Fan J. Sun Z. Wang Y. (2022). The assembly of a noncanonical LINC complex in *Saccharomyces cerevisiae* . Curr. Genet. 68 (1), 91–96. 10.1007/s00294-021-01220-0 34779871

[B40] Folco H. D. Chalamcharla V. R. Sugiyama T. Thillainadesan G. Zofall M. Balachandran V. (2017). Untimely expression of gametogenic genes in vegetative cells causes uniparental disomy. Nature 543 (7643), 126–130. 10.1038/nature21372 28199302 PMC5567995

[B41] Fowler K. R. Gutierrez-Velasco S. Martin-Castellanos C. Smith G. R. (2013). Protein determinants of meiotic DNA break hot spots. Mol. Cell 49 (5), 983–996. 10.1016/j.molcel.2013.01.008 23395004 PMC3595357

[B42] Fujita I. Nishihara Y. Tanaka M. Tsujii H. Chikashige Y. Watanabe Y. (2012). Telomere-nuclear envelope dissociation promoted by Rap1 phosphorylation ensures faithful chromosome segregation. Curr. Biol. 22 (20), 1932–1937. 10.1016/j.cub.2012.08.019 22959349

[B43] Gallo-Fernandez M. Saugar I. Ortiz-Bazan M. A. Vazquez M. V. Tercero J. A. (2012). Cell cycle-dependent regulation of the nuclease activity of Mus81-Eme1/Mms4. Nucleic Acids Res. 40 (17), 8325–8335. 10.1093/nar/gks599 22730299 PMC3458551

[B44] Garcia-Luis J. Clemente-Blanco A. Aragon L. Machin F. (2014). Cdc14 targets the Holliday junction resolvase Yen1 to the nucleus in early anaphase. Cell Cycle 13 (9), 1392–1399. 10.4161/cc.28370 24626187 PMC4050137

[B45] Gray S. Santiago E. R. Chappie J. S. Cohen P. E. (2020). Cyclin N-terminal domain-containing-1 coordinates meiotic crossover formation with cell-cycle progression in a cyclin-independent manner. Cell Rep. 32 (1), 107858. 10.1016/j.celrep.2020.107858 32640224 PMC7341696

[B46] Grigaitis R. Ranjha L. Wild P. Kasaciunaite K. Ceppi I. Kissling V. (2020). Phosphorylation of the RecQ helicase Sgs1/BLM controls its DNA unwinding activity during meiosis and mitosis. Dev. Cell 53 (6), 706–723. e705. 10.1016/j.devcel.2020.05.016 32504558

[B47] Gutierrez-Escribano P. Nurse P. (2015). A single cyclin-CDK complex is sufficient for both mitotic and meiotic progression in fission yeast. Nat. Commun. 6, 6871. 10.1038/ncomms7871 25891897 PMC4411289

[B48] Harashima H. Dissmeyer N. Schnittger A. (2013). Cell cycle control across the eukaryotic kingdom. Trends Cell Biol. 23 (7), 345–356. 10.1016/j.tcb.2013.03.002 23566594

[B49] Harper L. Golubovskaya I. Cande W. Z. (2004). A bouquet of chromosomes. J. Cell Sci. 117 (18), 4025–4032. 10.1242/jcs.01363 15316078

[B50] Hassold T. Hall H. Hunt P. (2007). The origin of human aneuploidy: Where we have been, where we are going. Hum. Mol. Genet. 16 (2), R203–R208. 10.1093/hmg/ddm243 17911163

[B51] Haversat J. Woglar A. Klatt K. Akerib C. C. Roberts V. Chen S. Y. (2022). Robust designation of meiotic crossover sites by CDK-2 through phosphorylation of the MutSγ complex. Proc. Natl. Acad. Sci. U. S. A. 119 (21), e2117865119. 10.1073/pnas.2117865119 35576467 PMC9173770

[B52] Henderson K. A. Kee K. Maleki S. Santini P. A. Keeney S. (2006). Cyclin-dependent kinase directly regulates initiation of meiotic recombination. Cell 125 (7), 1321–1332. 10.1016/j.cell.2006.04.039 16814718 PMC1950680

[B53] Hiraoka Y. Dernburg A. F. (2009). The SUN rises on meiotic chromosome dynamics. Dev. Cell 17 (5), 598–605. 10.1016/j.devcel.2009.10.014 19922865

[B54] Holloway J. K. Sun X. Yokoo R. Villeneuve A. M. Cohen P. E. (2014). Mammalian CNTD1 is critical for meiotic crossover maturation and deselection of excess precrossover sites. J. Cell Biol. 205 (5), 633–641. 10.1083/jcb.201401122 24891606 PMC4050721

[B55] Hu C. Inoue H. Sun W. Takeshita Y. Huang Y. Xu Y. (2019). Structural insights into chromosome attachment to the nuclear envelope by an inner nuclear membrane protein Bqt4 in fission yeast. Nucleic Acids Res. 47 (3), 1573–1584. 10.1093/nar/gky1186 30462301 PMC6379675

[B56] Huang X. Y. Niu J. Sun M. X. Zhu J. Gao J. F. Yang J. (2013). CYCLIN-DEPENDENT KINASE G1 is associated with the spliceosome to regulate CALLOSE SYNTHASE5 splicing and pollen wall formation in Arabidopsis. Plant Cell 25 (2), 637–648. 10.1105/tpc.112.107896 23404887 PMC3608783

[B57] Hunt P. Hassold T. (2010). Female meiosis: Coming unglued with age. Curr. Biol. 20 (17), R699–R702. 10.1016/j.cub.2010.08.011 20833308

[B58] Hunter N. Kleckner N. (2001). The single-end invasion: An asymmetric intermediate at the double-strand break to double-holliday junction transition of meiotic recombination. Cell 106 (1), 59–70. 10.1016/s0092-8674(01)00430-5 11461702

[B59] Hunter N. (2015). Meiotic recombination: The essence of heredity. Cold Spring Harb. Perspect. Biol. 7 (12), a016618. 10.1101/cshperspect.a016618 26511629 PMC4665078

[B60] Huttlin E. L. Jedrychowski M. P. Elias J. E. Goswami T. Rad R. Beausoleil S. A. (2010). A tissue-specific atlas of mouse protein phosphorylation and expression. Cell 143 (7), 1174–1189. 10.1016/j.cell.2010.12.001 21183079 PMC3035969

[B61] Ishiguro T. Tanaka K. Sakuno T. Watanabe Y. (2010). Shugoshin-PP2A counteracts casein-kinase-1-dependent cleavage of Rec8 by separase. Nat. Cell Biol. 12 (5), 500–506. 10.1038/ncb2052 20383139

[B62] Kar F. M. Hochwagen A. (2021). Phospho-regulation of meiotic prophase. Front. Cell Dev. Biol. 9, 667073. 10.3389/fcell.2021.667073 33928091 PMC8076904

[B63] Karaiskou A. Perez L. H. Ferby I. Ozon R. Jessus C. Nebreda A. R. (2001). Differential regulation of Cdc2 and Cdk2 by RINGO and cyclins. J. Biol. Chem. 276 (38), 36028–36034. 10.1074/jbc.M104722200 11461916

[B64] Kciuk M. Gielecinska A. Mujwar S. Mojzych M. Kontek R. (2022). Cyclin-dependent kinases in DNA damage response. Biochim. Biophys. Acta. Rev. Cancer 1877 (3), 188716. 10.1016/j.bbcan.2022.188716 35271993

[B65] Keeney S. Giroux C. N. Kleckner N. (1997). Meiosis-specific DNA double-strand breaks are catalyzed by Spo11, a member of a widely conserved protein family. Cell 88 (3), 375–384. 10.1016/s0092-8674(00)81876-0 9039264

[B66] Keeney S. Lange J. Mohibullah N. (2014). Self-organization of meiotic recombination initiation: General principles and molecular pathways. Annu. Rev. Genet. 48, 187–214. 10.1146/annurev-genet-120213-092304 25421598 PMC4291115

[B67] Kim Y. Kostow N. Dernburg A. F. (2015). The chromosome Axis mediates feedback control of CHK-2 to ensure crossover formation in *C. elegans* . Dev. Cell 35 (2), 247–261. 10.1016/j.devcel.2015.09.021 26506311 PMC4624198

[B68] Klutstein M. Cooper J. P. (2014). The Chromosomal Courtship Dance-homolog pairing in early meiosis. Curr. Opin. Cell Biol. 26, 123–131. 10.1016/j.ceb.2013.12.004 24529254 PMC6329632

[B69] Klutstein M. Fennell A. Fernandez-Alvarez A. Cooper J. P. (2015). The telomere bouquet regulates meiotic centromere assembly. Nat. Cell Biol. 17 (4), 458–469. 10.1038/ncb3132 25774833 PMC7682571

[B70] Koszul R. Kleckner N. (2009). Dynamic chromosome movements during meiosis: A way to eliminate unwanted connections? Trends Cell Biol. 19 (12), 716–724. 10.1016/j.tcb.2009.09.007 19854056 PMC2787882

[B71] Kulkarni D. S. Owens S. N. Honda M. Ito M. Yang Y. Corrigan M. W. (2020). PCNA activates the MutLγ endonuclease to promote meiotic crossing over. Nature 586 (7830), 623–627. 10.1038/s41586-020-2645-6 32814343 PMC8284803

[B72] Lai Y. J. Lin F. M. Chuang M. J. Shen H. J. Wang T. F. (2011). Genetic requirements and meiotic function of phosphorylation of the yeast axial element protein Red1. Mol. Cell. Biol. 31 (5), 912–923. 10.1128/MCB.00895-10 21173162 PMC3067829

[B73] Lam I. Keeney S. (2014). Mechanism and regulation of meiotic recombination initiation. Cold Spring Harb. Perspect. Biol. 7 (1), a016634. 10.1101/cshperspect.a016634 25324213 PMC4292169

[B74] Li H. Chen H. Zhang X. Qi Y. Wang B. Cui Y. (2022). Global phosphoproteomic analysis identified key kinases regulating male meiosis in mouse. Cell. Mol. Life Sci. 79 (8), 467. 10.1007/s00018-022-04507-8 35930080 PMC11071816

[B75] Li J. Qian W. P. Sun Q. Y. (2019). Cyclins regulating oocyte meiotic cell cycle progression. Biol. Reprod. 101 (5), 878–881. 10.1093/biolre/ioz143 31347666 PMC6877757

[B76] Link J. Jahn D. Alsheimer M. (2015). Structural and functional adaptations of the mammalian nuclear envelope to meet the meiotic requirements. Nucleus 6 (2), 93–101. 10.1080/19491034.2015.1004941 25674669 PMC4615672

[B77] Link J. Leubner M. Schmitt J. Gob E. Benavente R. Jeang K. T. (2014). Analysis of meiosis in SUN1 deficient mice reveals a distinct role of SUN2 in mammalian meiotic LINC complex formation and function. PLoS Genet. 10 (2), e1004099. 10.1371/journal.pgen.1004099 24586178 PMC3937131

[B78] Liu W. Wang L. Zhao W. Song G. Xu R. Wang G. (2014). Phosphorylation of CDK2 at threonine 160 regulates meiotic pachytene and diplotene progression in mice. Dev. Biol. 392 (1), 108–116. 10.1016/j.ydbio.2014.04.018 24797635

[B79] Lorenz A. Wells J. L. Pryce D. W. Novatchkova M. Eisenhaber F. McFarlane R. J. (2004). *S. pombe* meiotic linear elements contain proteins related to synaptonemal complex components. J. Cell Sci. 117 (15), 3343–3351. 10.1242/jcs.01203 15226405

[B80] MacKenzie A. M. Lacefield S. (2020). CDK regulation of meiosis: Lessons from *S. cerevisiae* and *S. pombe* . Genes (Basel) 11 (7), E723. 10.3390/genes11070723 PMC7397238 32610611

[B81] MacQueen A. J. Villeneuve A. M. (2001). Nuclear reorganization and homologous chromosome pairing during meiotic prophase require *C. elegans* chk-2. Genes Dev. 15 (13), 1674–1687. 10.1101/gad.902601 11445542 PMC312723

[B82] Malapeira J. Moldon A. Hidalgo E. Smith G. R. Nurse P. Ayte J. (2005). A meiosis-specific cyclin regulated by splicing is required for proper progression through meiosis. Mol. Cell. Biol. 25 (15), 6330–6337. 10.1128/MCB.25.15.6330-6337.2005 16024772 PMC1190344

[B83] Malumbres M. (2014). Cyclin-dependent kinases. Genome Biol. 15 (6), 122. 10.1186/gb4184 25180339 PMC4097832

[B84] Manterola M. Sicinski P. Wolgemuth D. J. (2016). E-type cyclins modulate telomere integrity in mammalian male meiosis. Chromosoma 125 (2), 253–264. 10.1007/s00412-015-0564-3 26712234 PMC4833587

[B85] Marston A. L. Amon A. (2004). Meiosis: Cell-cycle controls shuffle and deal. Nat. Rev. Mol. Cell Biol. 5 (12), 983–997. 10.1038/nrm1526 15573136

[B86] Martin-Castellanos C. Fowler K. R. Smith G. R. (2013). Making chromosomes hot for breakage. Cell Cycle 12 (9), 1327–1328. 10.4161/cc.24576 23588069 PMC3674054

[B87] Martinerie L. Manterola M. Chung S. S. Panigrahi S. K. Weisbach M. Vasileva A. (2014). Mammalian E-type cyclins control chromosome pairing, telomere stability and CDK2 localization in male meiosis. PLoS Genet. 10 (2), e1004165. 10.1371/journal.pgen.1004165 24586195 PMC3937215

[B88] Matos J. Blanco M. G. Maslen S. Skehel J. M. West S. C. (2011). Regulatory control of the resolution of DNA recombination intermediates during meiosis and mitosis. Cell 147 (1), 158–172. 10.1016/j.cell.2011.08.032 21962513 PMC3560330

[B89] Matos J. Blanco M. G. West S. C. (2013). Cell-cycle kinases coordinate the resolution of recombination intermediates with chromosome segregation. Cell Rep. 4 (1), 76–86. 10.1016/j.celrep.2013.05.039 23810555

[B90] Mikolcevic P. Isoda M. Shibuya H. del Barco Barrantes I. Igea A. Suja J. A. (2016). Essential role of the Cdk2 activator RingoA in meiotic telomere tethering to the nuclear envelope. Nat. Commun. 7, 11084. 10.1038/ncomms11084 27025256 PMC4820962

[B91] Miyoshi T. Ito M. Kugou K. Yamada S. Furuichi M. Oda A. (2012). A central coupler for recombination initiation linking chromosome architecture to S phase checkpoint. Mol. Cell 47 (5), 722–733. 10.1016/j.molcel.2012.06.023 22841486

[B92] Moiseeva V. Amelina H. Collopy L. C. Armstrong C. A. Pearson S. R. Tomita K. (2017). The telomere bouquet facilitates meiotic prophase progression and exit in fission yeast. Cell Discov. 3, 17041. 10.1038/celldisc.2017.41 29123917 PMC5674143

[B93] Moldon A. Malapeira J. Gabrielli N. Gogol M. Gomez-Escoda B. Ivanova T. (2008). Promoter-driven splicing regulation in fission yeast. Nature 455 (7215), 997–1000. 10.1038/nature07325 18815595

[B94] Molnar M. Doll E. Yamamoto A. Hiraoka Y. Kohli J. (2003). Linear element formation and their role in meiotic sister chromatid cohesion and chromosome pairing. J. Cell Sci. 116 (9), 1719–1731. 10.1242/jcs.00387 12665553

[B95] Morgan D. O. (1995). Principles of CDK regulation. Nature 374 (6518), 131–134. 10.1038/374131a0 7877684

[B96] Murakami H. Keeney S. (2008). Regulating the formation of DNA double-strand breaks in meiosis. Genes Dev. 22 (3), 286–292. 10.1101/gad.1642308 18245442 PMC2731648

[B97] Murakami H. Keeney S. (2014). Temporospatial coordination of meiotic DNA replication and recombination via DDK recruitment to replisomes. Cell 158 (4), 861–873. 10.1016/j.cell.2014.06.028 25126790 PMC4141489

[B98] Nagaoka S. I. Hassold T. J. Hunt P. A. (2012). Human aneuploidy: Mechanisms and new insights into an age-old problem. Nat. Rev. Genet. 13 (7), 493–504. 10.1038/nrg3245 22705668 PMC3551553

[B99] Nambiar M. Smith G. R. (2018). Pericentromere-specific cohesin complex prevents meiotic pericentric DNA double-strand breaks and lethal crossovers. Mol. Cell 71 (4), 540–553. e544. 10.1016/j.molcel.2018.06.035 30078721 PMC6097939

[B100] Ogino K. Hirota K. Matsumoto S. Takeda T. Ohta K. Arai K. (2006). Hsk1 kinase is required for induction of meiotic dsDNA breaks without involving checkpoint kinases in fission yeast. Proc. Natl. Acad. Sci. U. S. A. 103 (21), 8131–8136. 10.1073/pnas.0602498103 16698922 PMC1472441

[B101] Ogino K. Masai H. (2006). Rad3-Cds1 mediates coupling of initiation of meiotic recombination with DNA replication. Mei4-dependent transcription as a potential target of meiotic checkpoint. J. Biol. Chem. 281 (3), 1338–1344. 10.1074/jbc.M505767200 16286472

[B102] Ortega S. Prieto I. Odajima J. Martin A. Dubus P. Sotillo R. (2003). Cyclin-dependent kinase 2 is essential for meiosis but not for mitotic cell division in mice. Nat. Genet. 35 (1), 25–31. 10.1038/ng1232 12923533

[B103] Padmore R. Cao L. Kleckner N. (1991). Temporal comparison of recombination and synaptonemal complex formation during meiosis in *S. cerevisiae* . Cell 66 (6), 1239–1256. 10.1016/0092-8674(91)90046-2 1913808

[B104] Palmer N. Talib S. Z. A. Kaldis P. (2019). Diverse roles for CDK-associated activity during spermatogenesis. FEBS Lett. 593 (20), 2925–2949. 10.1002/1873-3468.13627 31566717 PMC6900092

[B105] Palmer N. Talib S. Z. A. Singh P. Goh C. M. F. Liu K. Schimenti J. C. (2020). A novel function for CDK2 activity at meiotic crossover sites. PLoS Biol. 18 (10), e3000903. 10.1371/journal.pbio.3000903 33075054 PMC7595640

[B106] Panizza S. Mendoza M. A. Berlinger M. Huang L. Nicolas A. Shirahige K. (2011). Spo11-accessory proteins link double-strand break sites to the chromosome axis in early meiotic recombination. Cell 146 (3), 372–383. 10.1016/j.cell.2011.07.003 21816273

[B107] Patel J. T. Bottrill A. Prosser S. L. Jayaraman S. Straatman K. Fry A. M. (2014). Mitotic phosphorylation of SUN1 loosens its connection with the nuclear lamina while the LINC complex remains intact. Nucleus 5 (5), 462–473. 10.4161/nucl.36232 25482198 PMC4164488

[B108] Payliss B. J. Tse Y. W. E. Reichheld S. E. Lemak A. Yun H. Y. Houliston S. (2022). Phosphorylation of the DNA repair scaffold SLX4 drives folding of the SAP domain and activation of the MUS81-EME1 endonuclease. Cell Rep. 41 (4), 111537. 10.1016/j.celrep.2022.111537 36288699

[B109] Penkner A. M. Fridkin A. Gloggnitzer J. Baudrimont A. Machacek T. Woglar A. (2009). Meiotic chromosome homology search involves modifications of the nuclear envelope protein Matefin/SUN-1. Cell 139 (5), 920–933. 10.1016/j.cell.2009.10.045 19913286

[B110] Petronczki M. Siomos M. F. Nasmyth K. (2003). Un menage a quatre: The molecular biology of chromosome segregation in meiosis. Cell 112 (4), 423–440. 10.1016/s0092-8674(03)00083-7 12600308

[B111] Phadnis N. Cipak L. Polakova S. Hyppa R. W. Cipakova I. Anrather D. (2015). Casein kinase 1 and phosphorylation of cohesin subunit Rec11 (SA3) promote meiotic recombination through linear element formation. PLoS Genet. 11 (5), e1005225. 10.1371/journal.pgen.1005225 25993311 PMC4439085

[B112] Polakova S. Molnarova L. Hyppa R. W. Benko Z. Misova I. Schleiffer A. (2016). Dbl2 regulates Rad51 and DNA joint molecule metabolism to ensure proper meiotic chromosome segregation. PLoS Genet. 12 (6), e1006102. 10.1371/journal.pgen.1006102 27304859 PMC4909299

[B113] Prasada Rao H. B. Sato T. Challa K. Fujita Y. Shinohara M. Shinohara A. (2021). Phosphorylation of luminal region of the SUN-domain protein Mps3 promotes nuclear envelope localization during meiosis. Elife 10, e63119. 10.7554/eLife.63119 34586062 PMC8570693

[B114] Qiao H. Prasada Rao H. B. Yang Y. Fong J. H. Cloutier J. M. Deacon D. C. (2014). Antagonistic roles of ubiquitin ligase HEI10 and SUMO ligase RNF212 regulate meiotic recombination. Nat. Genet. 46 (2), 194–199. 10.1038/ng.2858 24390283 PMC4356240

[B115] Reynolds A. Qiao H. Yang Y. Chen J. K. Jackson N. Biswas K. (2013). RNF212 is a dosage-sensitive regulator of crossing-over during mammalian meiosis. Nat. Genet. 45 (3), 269–278. 10.1038/ng.2541 23396135 PMC4245152

[B116] Robert T. Nore A. Brun C. Maffre C. Crimi B. Bourbon H. M. (2016). The TopoVIB-Like protein family is required for meiotic DNA double-strand break formation. Science 351 (6276), 943–949. 10.1126/science.aad5309 26917764

[B117] Rosu S. Zawadzki K. A. Stamper E. L. Libuda D. E. Reese A. L. Dernburg A. F. (2013). The *C. elegans* DSB-2 protein reveals a regulatory network that controls competence for meiotic DSB formation and promotes crossover assurance. PLoS Genet. 9 (8), e1003674. 10.1371/journal.pgen.1003674 23950729 PMC3738457

[B118] Rumpf C. Cipak L. Dudas A. Benko Z. Pozgajova M. Riedel C. G. (2010). Casein kinase 1 is required for efficient removal of Rec8 during meiosis I. Cell Cycle 9 (13), 2657–2662. 10.4161/cc.9.13.12146 20581463 PMC3083834

[B119] Sakuno T. Watanabe Y. (2009). Studies of meiosis disclose distinct roles of cohesion in the core centromere and pericentromeric regions. Chromosome Res. 17 (2), 239–249. 10.1007/s10577-008-9013-y 19308704

[B120] San-Segundo P. A. Clemente-Blanco A. (2020). Resolvases, dissolvases, and helicases in homologous recombination: Clearing the road for chromosome segregation. Genes (Basel) 11 (1), E71. 10.3390/genes11010071 PMC7017083 31936378

[B121] Scherthan H. (2007). Telomere attachment and clustering during meiosis. Cell. Mol. Life Sci. 64 (2), 117–124. 10.1007/s00018-006-6463-2 17219025 PMC11136177

[B122] Shibuya H. Hernandez-Hernandez A. Morimoto A. Negishi L. Hoog C. Watanabe Y. (2015). MAJIN links telomeric DNA to the nuclear membrane by exchanging telomere cap. Cell 163 (5), 1252–1266. 10.1016/j.cell.2015.10.030 26548954

[B123] Shibuya H. Ishiguro K. Watanabe Y. (2014). The TRF1-binding protein TERB1 promotes chromosome movement and telomere rigidity in meiosis. Nat. Cell Biol. 16 (2), 145–156. 10.1038/ncb2896 24413433

[B124] Shibuya H. Watanabe Y. (2014). The meiosis-specific modification of mammalian telomeres. Cell Cycle 13 (13), 2024–2028. 10.4161/cc.29350 24870409 PMC4111693

[B125] Sou I. F. Hamer G. Tee W.-W. Vader G. McClurg U. L. (2022). Cancer and meiotic gene expression: Two sides of the same coin? Curr. Top. Dev. Biol. E-pub ahead of print. 10.1016/bs.ctdb.2022.06.002 36681477

[B126] Spirek M. Estreicher A. Csaszar E. Wells J. McFarlane R. J. Watts F. Z. (2010). SUMOylation is required for normal development of linear elements and wild-type meiotic recombination in *Schizosaccharomyces pombe* . Chromosoma 119 (1), 59–72. 10.1007/s00412-009-0241-5 19756689

[B127] Stamper E. L. Rodenbusch S. E. Rosu S. Ahringer J. Villeneuve A. M. Dernburg A. F. (2013). Identification of DSB-1, a protein required for initiation of meiotic recombination in *Caenorhabditis elegans*, illuminates a crossover assurance checkpoint. PLoS Genet. 9 (8), e1003679. 10.1371/journal.pgen.1003679 23990794 PMC3749324

[B128] Stern B. Nurse P. (1996). A quantitative model for the cdc2 control of S phase and mitosis in fission yeast. Trends Genet. 12 (9), 345–350. 10.1016/s0168-9525(96)80016-3 8855663

[B129] Sun W. Lorenz A. Osman F. Whitby M. C. (2011). A failure of meiotic chromosome segregation in a fbh1Delta mutant correlates with persistent Rad51-DNA associations. Nucleic Acids Res. 39 (5), 1718–1731. 10.1093/nar/gkq977 21149262 PMC3061084

[B130] Swaffer M. P. Jones A. W. Flynn H. R. Snijders A. P. Nurse P. (2016). CDK substrate phosphorylation and ordering the cell cycle. Cell 167 (7), 1750–1761. e1716. 10.1016/j.cell.2016.11.034 27984725 PMC5161751

[B131] Szakal B. Branzei D. (2013). Premature Cdk1/Cdc5/Mus81 pathway activation induces aberrant replication and deleterious crossover. EMBO J. 32 (8), 1155–1167. 10.1038/emboj.2013.67 23531881 PMC3630363

[B132] Tang X. Jin Y. Cande W. Z. (2006). Bqt2p is essential for initiating telomere clustering upon pheromone sensing in fission yeast. J. Cell Biol. 173 (6), 845–851. 10.1083/jcb.200602152 16769823 PMC2063910

[B133] Toby G. G. Gherraby W. Coleman T. R. Golemis E. A. (2003). A novel RING finger protein, human enhancer of invasion 10, alters mitotic progression through regulation of cyclin B levels. Mol. Cell. Biol. 23 (6), 2109–2122. 10.1128/MCB.23.6.2109-2122.2003 12612082 PMC149478

[B134] Tomita K. Cooper J. P. (2006). The meiotic chromosomal bouquet: SUN collects flowers. Cell 125 (1), 19–21. 10.1016/j.cell.2006.03.020 16615884

[B135] Tomita K. Cooper J. P. (2007). The telomere bouquet controls the meiotic spindle. Cell 130 (1), 113–126. 10.1016/j.cell.2007.05.024 17632059

[B136] Tonami Y. Murakami H. Shirahige K. Nakanishi M. (2005). A checkpoint control linking meiotic S phase and recombination initiation in fission yeast. Proc. Natl. Acad. Sci. U. S. A. 102 (16), 5797–5801. 10.1073/pnas.0407236102 15805194 PMC556284

[B137] Trovesi C. Manfrini N. Falcettoni M. Longhese M. P. (2013). Regulation of the DNA damage response by cyclin-dependent kinases. J. Mol. Biol. 425 (23), 4756–4766. 10.1016/j.jmb.2013.04.013 23603016

[B138] Tu Z. Bayazit M. B. Liu H. Zhang J. Busayavalasa K. Risal S. (2017). Speedy A-Cdk2 binding mediates initial telomere-nuclear envelope attachment during meiotic prophase I independent of Cdk2 activation. Proc. Natl. Acad. Sci. U. S. A. 114 (3), 592–597. 10.1073/pnas.1618465114 28031483 PMC5255603

[B139] Tuna M. Knuutila S. Mills G. B. (2009). Uniparental disomy in cancer. Trends Mol. Med. 15 (3), 120–128. 10.1016/j.molmed.2009.01.005 19246245

[B140] Ubersax J. A. Woodbury E. L. Quang P. N. Paraz M. Blethrow J. D. Shah K. (2003). Targets of the cyclin-dependent kinase Cdk1. Nature 425 (6960), 859–864. 10.1038/nature02062 14574415

[B141] Uhlmann F. Bouchoux C. Lopez-Aviles S. (2011). A quantitative model for cyclin-dependent kinase control of the cell cycle: Revisited. Philos. Trans. R. Soc. Lond. B Biol. Sci. 366 (1584), 3572–3583. 10.1098/rstb.2011.0082 22084384 PMC3203462

[B142] Viera A. Alsheimer M. Gomez R. Berenguer I. Ortega S. Symonds C. E. (2015). CDK2 regulates nuclear envelope protein dynamics and telomere attachment in mouse meiotic prophase. J. Cell Sci. 128 (1), 88–99. 10.1242/jcs.154922 25380821

[B143] Viera A. Rufas J. S. Martinez I. Barbero J. L. Ortega S. Suja J. A. (2009). CDK2 is required for proper homologous pairing, recombination and sex-body formation during male mouse meiosis. J. Cell Sci. 122 (12), 2149–2159. 10.1242/jcs.046706 19494131

[B144] Vrielynck N. Chambon A. Vezon D. Pereira L. Chelysheva L. De Muyt A. (2016). A DNA topoisomerase VI-like complex initiates meiotic recombination. Science 351 (6276), 939–943. 10.1126/science.aad5196 26917763

[B145] Wan L. Niu H. Futcher B. Zhang C. Shokat K. M. Boulton S. J. (2008). Cdc28-Clb5 (CDK-S) and Cdc7-Dbf4 (DDK) collaborate to initiate meiotic recombination in yeast. Genes Dev. 22 (3), 386–397. 10.1101/gad.1626408 18245450 PMC2216697

[B146] Wang G. Wu X. Zhou L. Gao S. Yun D. Liang A. (2020). Tethering of telomeres to the nuclear envelope is mediated by SUN1-MAJIN and possibly promoted by SPDYA-CDK2 during meiosis. Front. Cell Dev. Biol. 8, 845. 10.3389/fcell.2020.00845 33015044 PMC7509418

[B147] Ward J. O. Reinholdt L. G. Motley W. W. Niswander L. M. Deacon D. C. Griffin L. B. (2007). Mutation in mouse hei10, an e3 ubiquitin ligase, disrupts meiotic crossing over. PLoS Genet. 3 (8), e139. 10.1371/journal.pgen.0030139 17784788 PMC1959360

[B148] Wijnker E. Harashima H. Muller K. Parra-Nunez P. de Snoo C. B. van de Belt J. (2019). The Cdk1/Cdk2 homolog CDKA;1 controls the recombination landscape in Arabidopsis. Proc. Natl. Acad. Sci. U. S. A. 116 (25), 12534–12539. 10.1073/pnas.1820753116 31164422 PMC6589648

[B149] Woglar A. Daryabeigi A. Adamo A. Habacher C. Machacek T. La Volpe A. (2013). Matefin/SUN-1 phosphorylation is part of a surveillance mechanism to coordinate chromosome synapsis and recombination with meiotic progression and chromosome movement. PLoS Genet. 9 (3), e1003335. 10.1371/journal.pgen.1003335 23505384 PMC3591285

[B150] Wyatt H. D. Sarbajna S. Matos J. West S. C. (2013). Coordinated actions of SLX1-SLX4 and MUS81-EME1 for Holliday junction resolution in human cells. Mol. Cell 52 (2), 234–247. 10.1016/j.molcel.2013.08.035 24076221

[B151] Xu J. Li X. Song W. Wang W. Gao S. (2019). Cyclin Cyc2p is required for micronuclear bouquet formation in Tetrahymena thermophila. Sci. China. Life Sci. 62 (5), 668–680. 10.1007/s11427-018-9369-3 30820856

[B152] Yamamoto A. Hiraoka Y. (2001). How do meiotic chromosomes meet their homologous partners?: Lessons from fission yeast. Bioessays 23 (6), 526–533. 10.1002/bies.1072 11385632

[B153] Yang C. Sofroni K. Wijnker E. Hamamura Y. Carstens L. Harashima H. (2020). The Arabidopsis Cdk1/Cdk2 homolog CDKA;1 controls chromosome axis assembly during plant meiosis. EMBO J. 39 (3), e101625. 10.15252/embj.2019101625 31556459 PMC6996576

[B154] Yokoo R. Zawadzki K. A. Nabeshima K. Drake M. Arur S. Villeneuve A. M. (2012). COSA-1 reveals robust homeostasis and separable licensing and reinforcement steps governing meiotic crossovers. Cell 149 (1), 75–87. 10.1016/j.cell.2012.01.052 22464324 PMC3339199

[B155] Zakharyevich K. Tang S. Ma Y. Hunter N. (2012). Delineation of joint molecule resolution pathways in meiosis identifies a crossover-specific resolvase. Cell 149 (2), 334–347. 10.1016/j.cell.2012.03.023 22500800 PMC3377385

[B156] Zheng T. Nibau C. Phillips D. W. Jenkins G. Armstrong S. J. Doonan J. H. (2014). CDKG1 protein kinase is essential for synapsis and male meiosis at high ambient temperature in *Arabidopsis thaliana* . Proc. Natl. Acad. Sci. U. S. A. 111 (6), 2182–2187. 10.1073/pnas.1318460111 24469829 PMC3926089

[B157] Zhu Z. Mori S. Oshiumi H. Matsuzaki K. Shinohara M. Shinohara A. (2010). Cyclin-dependent kinase promotes formation of the synaptonemal complex in yeast meiosis. Genes cells. 15 (10), 1036–1050. 10.1111/j.1365-2443.2010.01440.x 20825495

[B158] Zickler D. Kleckner N. (2015). Recombination, pairing, and synapsis of homologs during meiosis. Cold Spring Harb. Perspect. Biol. 7 (6), a016626. 10.1101/cshperspect.a016626 25986558 PMC4448610

[B159] Zuela N. Gruenbaum Y. (2016). Matefin/SUN-1 phosphorylation on serine 43 is mediated by CDK-1 and required for its localization to centrosomes and normal mitosis in *C. elegans* embryos. Cells 5 (1), E8. 10.3390/cells5010008 PMC4810093 26927181

